# Enabling tumor-specific drug delivery by targeting the Warburg effect of cancer

**DOI:** 10.1016/j.xcrm.2024.101920

**Published:** 2025-01-13

**Authors:** Jian Zhang, Tony Pan, Jimmy Lee, Sanja Goldberg, Sarah Ann King, Erting Tang, Yifei Hu, Lifeng Chen, Alex Hoover, Linyong Zhu, Oliver S. Eng, Benjamin Dekel, Jun Huang, Xiaoyang Wu

**Affiliations:** 1 Department of Molecular Biomedical Sciences, College of Veterinary Medicine, North Carolina State University, Raleigh, NC 27606, USA; 2 Ben May Department for Cancer Research, The University of Chicago, Chicago, IL 60637, USA; 3 Pritzker School of Molecular Engineering, The University of Chicago, Chicago, IL 60637, USA; 4 Graduate Institute of Pathology, College of Medicine, National Taiwan University, Taipei, Taiwan; 5 Pediatric Stem Cell Research Institute, Safra Children’s Hospital, Sheba Medical Center, Tel Aviv, Israel; 6 Key Laboratory for Advanced Materials and Joint International Research Laboratory of Precision Chemistry and Molecular Engineering, Feringa Nobel Prize Scientist Joint Research Center, School of Chemistry and Molecular Engineering, East China University of Science & Technology, Shanghai 200237, China; 7 Department of Surgery, University of California, Irvine, Orange, CA 92868, USA; 8 Division of Pediatric Nephrology and Pediatric Stem Cell Research Institute, Safra Children’s Hospital, Sheba Medical Center, Tel Hasomer, Sago Center for Regenerative Medicine, Faculty of Medicine, Tel Aviv University, Tel Aviv, Israel

**Keywords:** Warburg effect, tumor metabolism, lactate, drug delivery, nanoparticle, chemotherapy, immunotherapy

## Abstract

Metabolic reprogramming of tumor cells is an emerging hallmark of cancer. Among all the changes in cancer metabolism, increased glucose uptake and the accumulation of lactate under normoxic conditions (the “Warburg effect”) is a common feature of cancer cells. In this study, we develop a lactate-responsive drug delivery platform by targeting the Warburg effect. We design and test a gold/mesoporous silica Janus nanoparticle system as a gated drug carrier, in which the gold particles are functionalized with lactate oxidase and the silica particles are capped with α-cyclodextrin through surface arylboronate modification. In the presence of lactate, the lactate oxidase generates hydrogen peroxide, which induces the self-immolation reaction of arylboronate, leading to uncapping and drug release. Our results demonstrate greatly improved drug delivery specificity and therapeutic efficacy with this platform for the treatment of different cancers. Our findings present an effective approach for drug delivery by metabolic targeting of tumors.

## Highlights

• The Warburg effect (lactate accumulation) is a key feature of cancer cells
• A lactate-responsive drug delivery system is developed to target this feature
• It uses Janus nanoparticles with lactate-triggered drug release
• It improves drug delivery specificity and effectiveness for cancer treatment

## Graphical abstract

**Figure:**
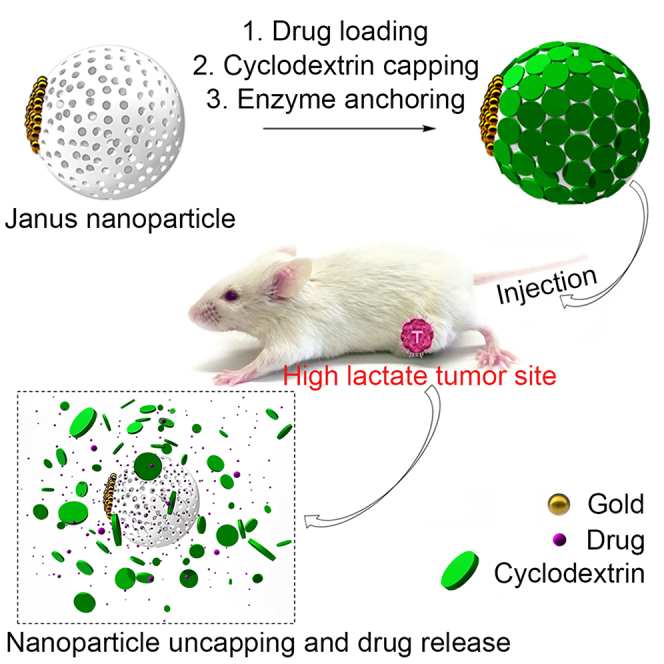

## Teaser

Zhang et al. develop a lactate-responsive drug delivery platform targeting the Warburg effect of cancer. With Janus nanoparticles, drugs can be released by elevated lactate levels in the tumor microenvironment, enhancing delivery specificity and therapeutic efficacy. This system provides a promising approach for targeted cancer therapy by leveraging cancer metabolism.

## Introduction

The effectiveness of cancer therapies is limited by drug accessibility to tumor tissues and undesired toxicity to healthy tissues.^1^ The success of cancer treatment is thus critically dependent on the effective and specific delivery of the drug to the tumor, which can reduce the side effects while improving therapeutic efficacy.^1^^,^^2^ In the past decades, a better understanding of tumor biology, together with increased availability of new biomaterials, including liposomes, nanoparticles, organic carriers, and hydrogels, has led to the development of novel drug delivery techniques for cancer treatment.^3^ Compared to conventional therapies, passive drug carriers have taken advantage of the enhanced permeability and retention (EPR) effect in the tumor tissue and active cellular uptake of cancer cells.^4^ Active targeting approaches have also been developed by the conjugation of drug carriers with molecules that recognize and bind to cancer-specific antigens.^5^ However, compared to the plethora of many successful pre-clinical studies, only a few passively targeted carriers have been approved for clinical use, such as Doxil and Abraxane.^6^ Physiological barriers of solid tumors, tumor heterogeneity, and complexity of the tumor microenvironment have limited the clinical benefits of different drug delivery systems.^7^ Significant obstacles related to regulatory compliance and commercialization also represent major hurdles to bridge the bench-to-bedside gap and clinical translation of drug delivery systems for cancer treatment.^8^^,^^9^^,^^10^

Cancer development and progression are highly associated with the tumor microenvironment.^11^^,^^12^ As tumor cells continue to proliferate, the tumor increases in size with a profound remodeling of the tumor microenvironment that causes tumor metabolic reprogramming and aberrant cellular energetics, which are emerging hallmarks of cancer and potential therapeutic targets for cancer treatment.^13^ Among all the changes in cancer metabolism, increased glucose uptake and the accumulation of lactate under normoxic conditions (aerobic glycolysis or the “Warburg effect”) is a common feature of cancer cells.^14^^,^^15^^,^^16^^,^^17^^,^^18^ It is now well accepted that the Warburg effect is a consequence of aberrant cellular respiration, oncogenic mutations, and overexpression of glycolytic enzymes and metabolite transporters in tumor cells.^16^^,^^19^^,^^20^^,^^21^ Tumor lactate is considered as an important signaling molecule that regulates the behavior of cancer cells, tumor-stroma interaction, and immune responses.^22^ Tumor lactate concentration can serve as a biomarker to predict cancer progression, metastasis, and survival in patients.^19^^,^^20^^,^^21^^,^^23^

Aerobic glycolysis leads to dramatically increased lactate concentration in solid tumors.^24^ Whereas healthy tissues have a lactate concentration of ∼1 mM, tumor tissues contain much higher lactate concentrations ranging from 10 to 20 mM, and up to 40 mM.^19^^,^^20^ The accumulation of lactate can lead to reduced pH in the tumor microenvironment, which has been leveraged for the development of pH-responsive drug delivery platforms.^25^ However, many studies have shown that the Warburg effect is not necessarily associated with tissue acidosis, thus limiting their applicability *in vivo.*^26^^,^^27^^,^^28^ Current pH-responsive drug delivery systems usually require a strong acidic environment (pH < 5.0) to trigger sufficient drug release, whereas most tumor tissues are only weakly acidic (pH between 6.0 and 7.0).^29^^,^^30^^,^^31^^,^^32^ Additionally, there are normal organs with regions of low pH that are not associated with cancer, such as the kidney and white pulp in the spleen.^33^^,^^34^^,^^35^ Thus, a drug delivery system with a chemo-specific trigger that can respond to lactate could serve as a more specific and effective platform to target tumors while sparing normal tissues.

In this study, we developed a lactate-responsive drug carrier based on enzyme-assisted Janus mesoporous silica nanoparticles. In this platform, the Janus particles are functionalized with lactate oxidase and arylboronate derivatives as caps to shield the mesoporous silica particles. The lactate oxidase acts as a reader and signal transmitter of tumor lactate by catalyzing the oxidation of lactate to produce pyruvate and hydrogen peroxide. The production of hydrogen peroxide in tumor tissues can induce the self-immolation reaction of arylboronate,^36^^,^^37^ leading to uncapping of mesoporous silica particles, drug release, and killing of tumor cells. Our data demonstrate superior drug delivery specificity and efficacy of this platform *in vitro* and *in vivo* for treatment of different solid tumors. Together, our study unravels an effective drug delivery approach by metabolic targeting of tumors.

## Results

### Fabrication of enzyme-functionalized nanoparticles

**Figure 1:**
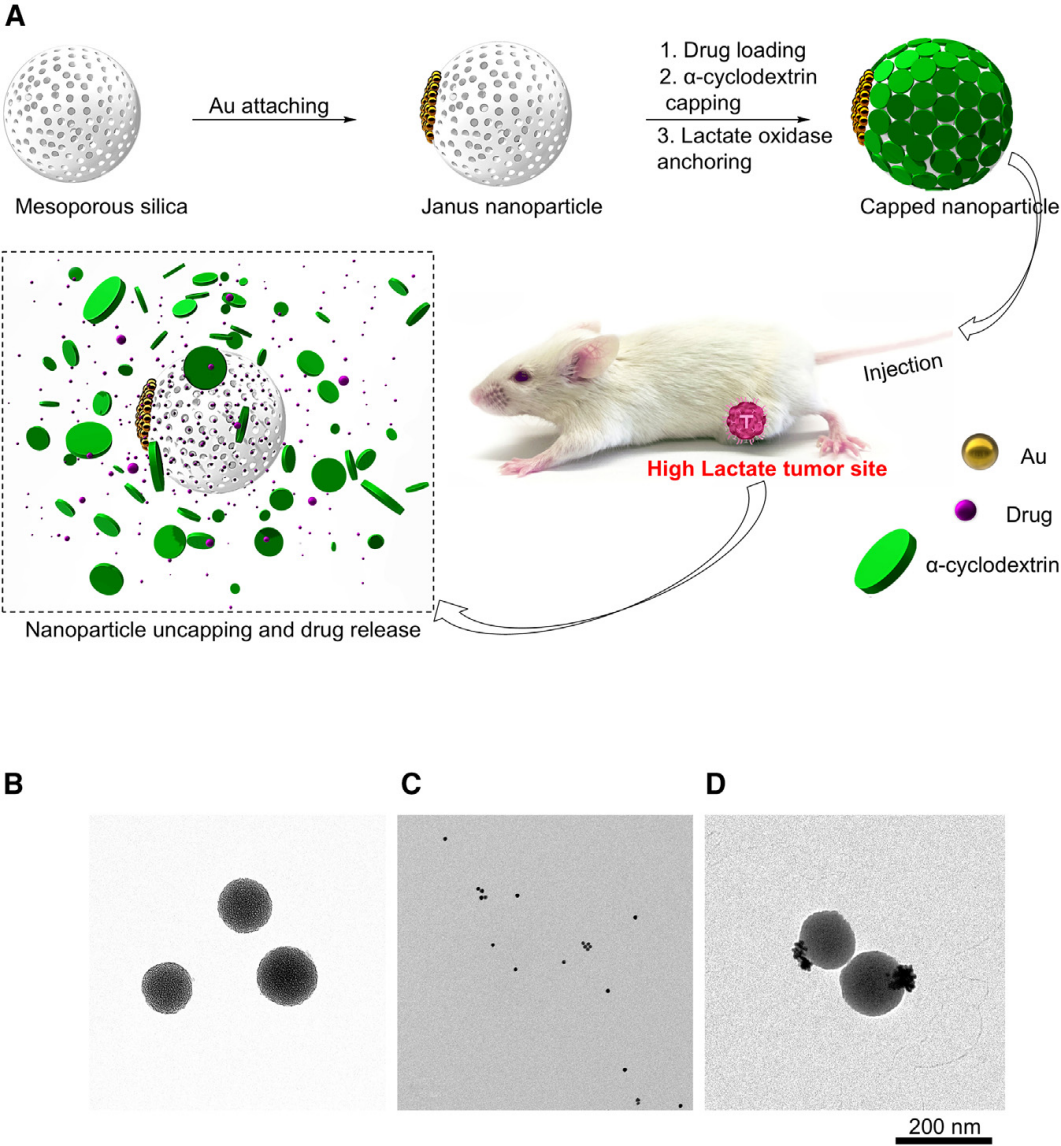
Development of the Janus nanoparticles for lactate-inducible drug release (A) Schematic illustration of the Janus nanoparticle fabrication for tumor-specific drug delivery. CD, α-cyclodextrin; Au, gold. (B–D) Representative TEM images of the mesoporous silica (B), Au (C) nanoparticles, and the Janus nanoparticles Au/silica (D).

To achieve lactate-inducible drug release, we chose surface-gated mesoporous silica nanoparticles because of their robust mechanical property, high loading capacity, and superior biocompatibility.^38^^,^^39^^,^^40^ However, it is challenging to develop a lactate-responsive gating mechanism as there are no chemical bonds that can selectively respond to lactate under physiological conditions. To address these issues, we designed an enzyme-functionalized Janus nanoparticle platform by leveraging the substrate specificity of lactate oxidase, which can catalyze the oxidation of lactate to produce pyruvate and hydrogen peroxide (Figure 1A; Figure S1A). The Janus particles are capped with α-cyclodextrin through arylboronic derivatives.^37^ Arylboronic esters are ideal responsive gating fragments because of their facile degradation mediated by hydrogen peroxide at physiological pH and temperature.^36^^,^^37^ Only in the presence of lactate, the production of hydrogen peroxide can induce self-immolation of arylboronate, leading to uncapping of nanoparticles and drug release.

To prepare the Janus nanoparticles, gold (Au) nanoparticles are produced based on the Turkevich-Frens method.^41^ Mesoporous silica particles (Figure S1A) are partially confined using paraffin wax.^42^ The exposed silica surface allows site-specific modification with thiol groups, which are used as linkers for the stable attachment of Au nanoparticles (Figures 1B–1D).^43^ The resulting Au/silica Janus nanoparticles are modified with arylboronate derivatives on the surface of silica nanoparticles. The modified nanoparticles are then loaded with drug and capped with α-cyclodextrin. The Au face of the Janus nanoparticles is further functionalized with a carboxyl group to allow the covalent immobilization of lactate oxidase by carbodiimide coupling reaction.^44^

The Janus nanoparticles have an average size of 129 ± 21 nm as determined by transmission electron microscopy (TEM) and dynamic light scattering (Figure S1B). The Janus nanoparticle is composed of a mesoporous silica nanoparticle (124 ± 18 nm) and around 20 ± 5 Au nanoparticles (4 ± 2 nm). After functionalization by the host-guest complexation between arylboronate derivative and α-cyclodextrin, the spherical morphology of the mesoporous silica nanoparticles is maintained, while the nanopores are obscured by the coating of α-cyclodextrin. The surface modifications of mesoporous silica nanoparticles are confirmed by the measurement of the surface ζ-potential, which measures the electrical potential difference between the dispersion medium and the stationary layer of fluid attached to the dispersed particle in a colloidal system (Figure S1C). Due to the protonation of the amino functionality, the aminopropyltriethoxysilane-modified Janus nanoparticles have a ζ-potential at 14 ± 2 mV. The ζ-potential is decreased to −3 ± 3 mV upon modification with arylboronate derivative and further decreased to −18 ± 4 mV after complexation with α-cyclodextrin. Fourier transform infrared (FT-IR) analysis determines the absorption of infrared radiation. FT-IR reveals that all Janus nanoparticles have the characteristic spectrum of siliceous materials with absorption bands at 460, 790, and 960 cm^−1^ (Figure S1D). The amide bond stretching in the Janus particles can be observed at 1,350 cm^−1^. After the coupling with α-cyclodextrin, the amide bond stretching is masked by the –C-H stretching from α-cyclodextrin. The conjugation of arylboronate derivatives and the coupling of α-cyclodextrin are also confirmed by thermogravimetric analyses (Figure S1E). The thermogravimetric analysis measures the mass change after heating of the sample, which can remove arylboronate derivative and α-cyclodextrin capping from the inorganic nanoparticles. The weight ratio of arylboronate derivatives and α-cyclodextrin on the surface of the nanoparticles is 8.7% and 7.1%. Enzymatic analysis reveals that ∼1.78 units of lactate oxidase are conjugated to 1 mg of Janus nanoparticles.

Lactate-inducible drug release can be achieved through other designs with silica nanoparticles functionalized with lactate oxidase. To examine this possibility, we covalently conjugated the α-cyclodextrin (capping molecules) with lactate oxidase before coating the mesoporous silica nanoparticles through arylboronate linkage (Figures S2A–S2C). TEM analyses confirm that the modified nanoparticles have an average size of 126 ± 19 nm (Figure S2D). After functionalization by modified α-cyclodextrin, the spherical morphology of the silica nanoparticle was maintained, while the nanopores were obscured by the α-cyclodextrin coating. Enzymatic analysis revealed that ∼3.11 units of lactate oxidase could be conjugated to 1 mg of Janus nanoparticles through this strategy.

### Lactate-inducible drug release of the Janus nanoparticles

**Figure 2:**
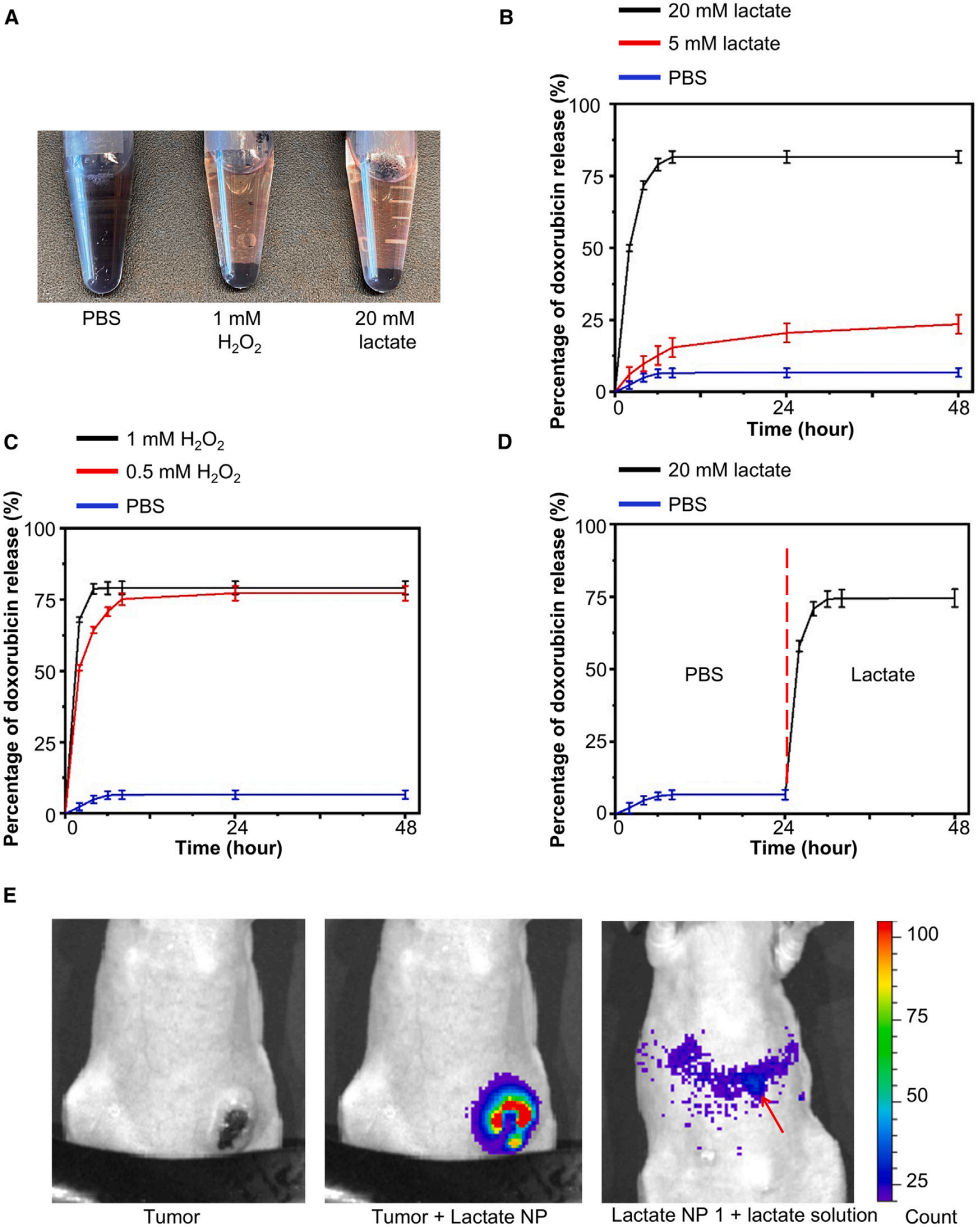
Lactate-responsive drug release from the Janus nanoparticles (A) Lactate can induce hydrogen peroxide-mediated uncapping and drug release of the Janus nanoparticles. After 24 h of incubation, the doxorubicin-loaded nanoparticles are presented as a stable purple colloid in phosphate-buffered saline solution (left), while the uncapped nanoparticles precipitate and doxorubicin is released to the supernatant in the presence of hydrogen peroxide (middle) or lactate (right). (B and C) Release kinetics of doxorubicin from the Janus nanoparticles at different concentrations of lactate (B) or H_2_O_2_ (C). *n* = 3. Data are presented as mean ± SD (standard deviation). All error bars represent SD. (D) Lactate can induce rapid release of doxorubicin from the Janus nanoparticles after 24 h incubation in PBS solution. *n* = 3. Data are presented as mean ± SD (standard deviation). (E) To monitor the potential uncapping and drug release *in vivo*, H_2_O_2_-responsive bioluminescence formulation was intraperitoneally injected to nude mice with 4T1 tumors. No luminescence signals were observed in mice without nanoparticle injection (control, left). Upon intravenous injection of the Janus nanoparticles, luminescence signals can be observed around the tumor (middle). Subcutaneous injection of lactate solution as a positive control also leads to luminescence signals around the injection site (arrow, right).

To evaluate the lactate responsiveness for drug delivery, the Janus nanoparticles were loaded with doxorubicin, a common chemotherapeutic drug used for breast cancer, sarcoma, lymphoma, and acute lymphocytic leukemia.^32^ The amount of loaded doxorubicin was ∼90 μmol per gram of nanoparticles (∼1.1 × 10^−16^ g of doxorubicin per nanoparticle). Importantly, the loaded nanoparticles were presented as a stable purple colloid in phosphate-buffered saline (PBS) solution after 24 h of incubation at 37°C. By contrast, in the presence of hydrogen peroxide or lactate, the nanoparticles precipitated, and loaded doxorubicin was released into the supernatant (Figure 2A). The uncapping of the α-cyclodextrin led to drug release and aggregation of mesoporous silica nanoparticles *in vitro*. The *in vitro* release kinetics were determined spectrophotometrically and showed slow release over 48 h in PBS, suggesting sufficient pore sealing by α-cyclodextrin. By contrast, the presence of hydrogen peroxide or lactate induced a rapid release of doxorubicin in a dose-dependent manner (Figures 2B and 2C). The addition of lactate solution 24 h after incubation in PBS initiated faster drug release, indicating the stability and inducibility of our lactate-responsive system *in vitro* (Figure 2D). Platinum-based drugs are common chemotherapeutic agents for many malignancies.^45^ Photosensitizer tris(bipyridine)ruthenium(II) chloride ([Ru(bpy)_3_]Cl_2_) has distinctive optical properties for potential cancer treatment.^46^^,^^47^ Our data demonstrate that cisplatin, oxaliplatin, and [Ru(bpy)_3_]Cl_2_ can be successfully loaded to the Janus nanoparticles and exhibit lactate-dependent release *in vitro* as doxorubicin (Figures S3A–S3C). The alternative strategy to prepare lactate oxidase-functionalized nanoparticles without Au (Figure S2) exhibits similar lactate-dependent drug release *in vitro* when loaded with doxorubicin (Figure S3D).

To examine the performance of our drug carrier *in vivo*, we used a peroxyoxalate-based nano-reactor formulation that can generate strong near-infrared signals in the presence of hydrogen peroxide.^33^ 4T1 mouse breast cancer cells were grafted subcutaneously to nude mice. We determined the lactate level in tumors and other organs. As expected, triple-negative breast cancer (TNBC) tumors have significantly elevated lactate levels (∼8.6 mM), whereas normal tissues such as muscle and heart have low levels of lactate (∼1 mM) (Figure S3E). The tumor-bearing nude mice were injected with peroxyoxalate imaging solution intraperitoneally, followed with intravenous injection of Au/mesoporous silica nanoparticles. Bright chemiluminescent signals can be detected around tumors shortly after the injection of nanoparticles, but not at other locations of the mice, strongly suggesting that our drug carrier can respond specifically to lactate and generate hydrogen peroxide at tumor tissues for local drug release as expected (Figure 2E).

### Enhanced drug release in tumors with the Janus nanoparticles

**Figure 3:**
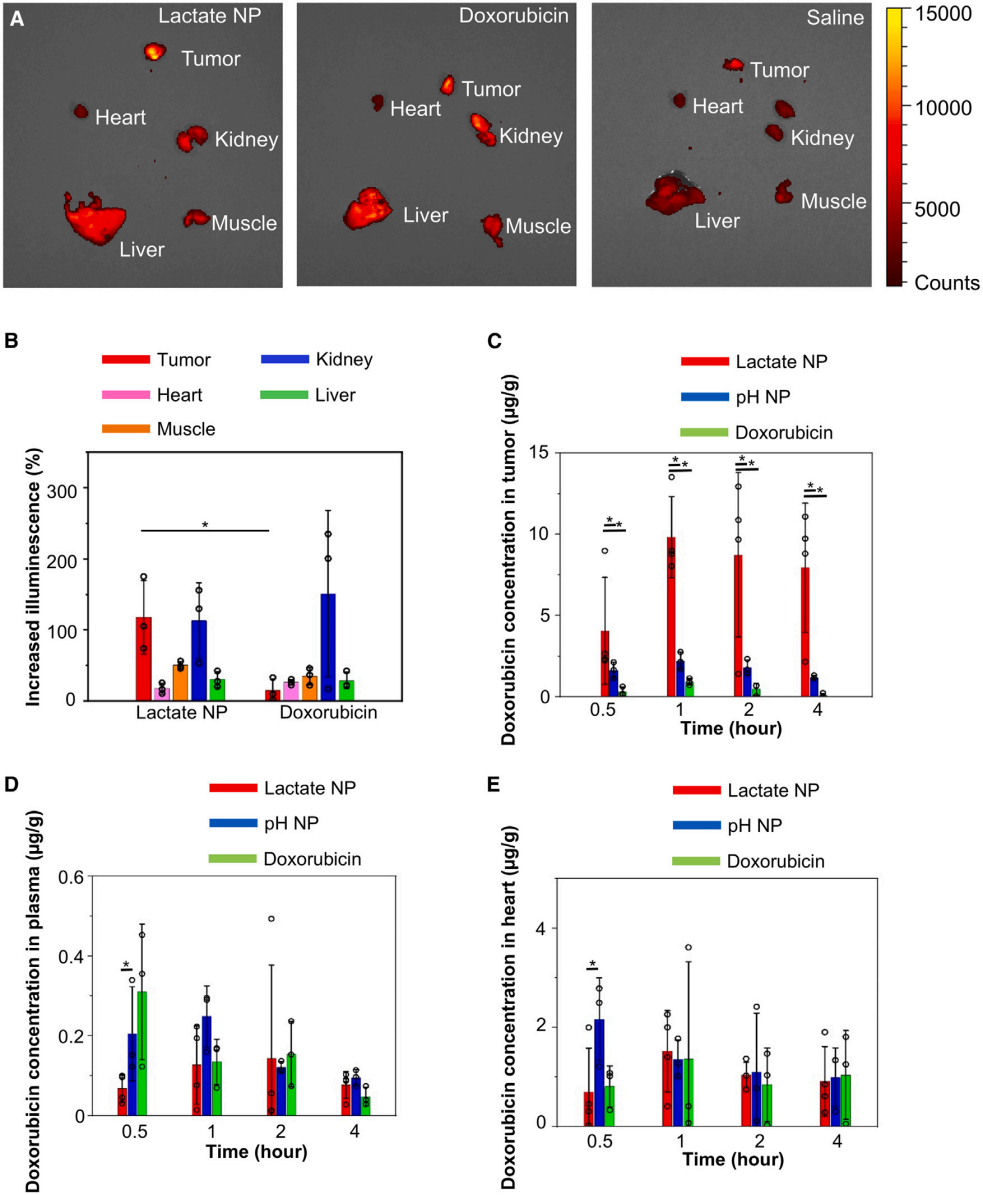
Enhanced tumor-specific drug delivery with the lactate-responsive nanocarrier (A and B) Fluorescence image (A) and quantification (B) of tumor and different organs after intravenous injection of saline (control) or doxorubicin with or without lactate-responsive nanoparticles (lactate NPs). *n* = 3. Data are presented as mean ± SD (standard deviation). All error bars represent SD. ∗: *p* < 0.05 (Student’s t test). (C–E) Quantification of doxorubicin distribution in tumor (C), plasma (D), or heart (E) after intravenous injection of free doxorubicin or doxorubicin within lactate-responsive nanoparticles or pH-responsive nanoparticles (pH NPs). The concentration of doxorubicin was evaluated by liquid chromatography/mass spectrometry. *n* = 3. Data are presented as mean ± SD (standard deviation). All error bars represent SD. ∗: *p* < 0.05 (Student’s t test).

To determine potential changes in drug biodistribution and pharmacokinetics, we prepared tumor-bearing mice by orthotopic injection of 4T1 cells to the fat pad of female BALB/c mice.^48^ As doxorubicin has strong intrinsic fluorescence, *ex vivo* fluorescence imaging provides an effective approach to monitor drug release upon lactate-response deliver. The distribution of doxorubicin in tumor and other major organs was examined by the *in vivo* imaging system 1 h after intravenous injection of the drug (free drug or within the lactate-responsive carriers) (Figure 3A).^49^^,^^50^^,^^51^^,^^52^^,^^53^ Quantification of the fluorescence signals showed a specific increase of doxorubicin distribution in tumors when delivered within the Janus nanoparticles, but not in other healthy organs, including heart, liver, muscle, and kidney (Figure 3B).

We further evaluated the doxorubicin release kinetics in different tissues by liquid chromatography/mass spectrometry (Figures 3C–3E and S3F–S3H).^54^^,^^55^^,^^56^ For comparison, we also prepared a typical pH-responsive nanocarrier for doxorubicin delivery (alginate/chitosan multilayer-coated mesoporous silica nanoparticles).^57^ Our results showed dramatically increased doxorubicin in tumors with the lactate-responsive particles. The peak concentration of doxorubicin increased by more than 10-fold when delivered with the Janus nanoparticles, compared with free drug injection. By contrast, the pH-responsive carrier had only a marginal effect in increasing the drug concentration in tumors. The free injection of doxorubicin had a peak concentration at 0.5 h in plasma, which was significantly reduced when delivered with the Janus nanoparticles (Figure 3D). A similar reduction was also observed for kidney and liver tissues. In both muscle and heart, delivery with the Janus nanoparticles had similar drug kinetics compared with free doxorubicin injection, but the concentration of doxorubicin was significantly lower than delivery with the pH-responsive particles at early time points (Figures 3E and S3F). Nanoparticles can be internalized via different endocytic pathways of cancer cells. Incubation of 4T1 cells with doxorubicin-loaded nanoparticles can lead to significant internalization of the particles (Figure S3I), as determined by confocal microscopy, suggesting that the nanoparticles can function both extracellularly and intracellularly. Together, our results demonstrated that the lactate-responsive drug carrier system can specifically increase drug delivery in tumors.

### Lactate-targeting drug delivery enhances therapeutic efficacy *in vivo*

**Figure 4:**
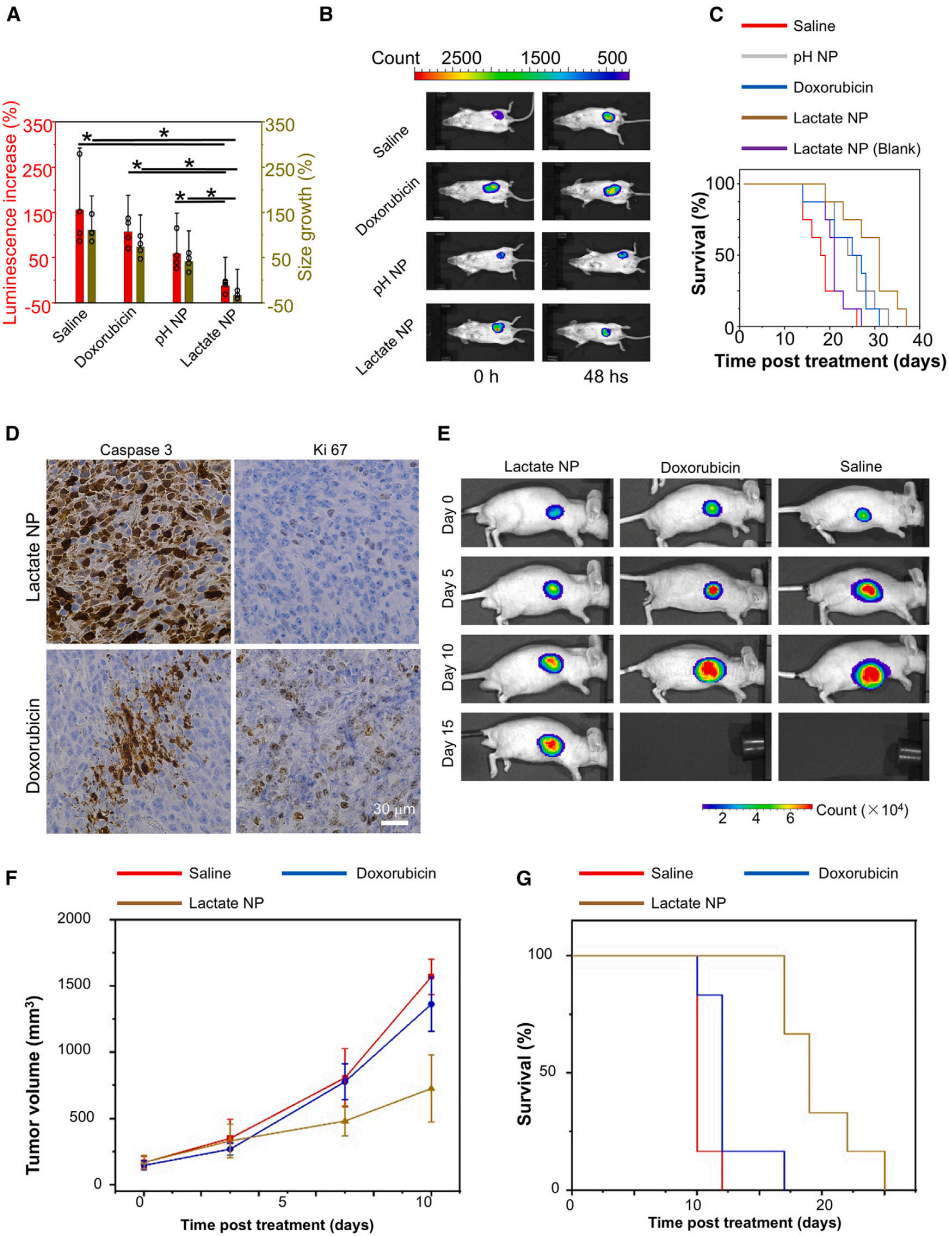
Lactate-responsive drug carriers enhance the therapeutic efficacy of chemotherapy *in vivo* (A) Change in tumor size after delivery of free doxorubicin or doxorubicin within lactate NPs or pH NPs. Injection of saline serves as a control. *n* = 4. Data are presented as mean ± SD. All error bars represent SD. ∗ represents *p* < 0.05 (Student’s t test). (B) 48 h after the treatment, delivery of the drug via the lactate-responsive nanoparticles, but not free doxorubicin or delivery with pH-responsive particles, leads to a marked reduction in tumor bioluminescence. (C) Kaplan-Meier survival curve of BALB/c mice with 4T1 tumors upon different treatment as indicated. *n* = 8. (D) Apoptosis and proliferation of tumor cells were determined by immunohistochemistry with different antibody as indicated. (E) The bioluminescence images of Ewing’s sarcoma upon different treatments as indicated. (F) Quantification of tumor size of Ewing’s sarcoma with different treatment. *n* = 6 independent samples. Data are presented as mean ± SD. All error bars represent SD. (G) Kaplan-Meier survival curve of mice with Ewing’s sarcoma upon different treatments as indicated. *n* = 6.

Doxorubicin is the first-line treatment for breast cancer.^58^ To determine the potential therapeutic efficacy of our tumor-specific drug delivery platform, we delivered doxorubicin with or without the lactate-responsive nanoparticle carrier at the same dose to animals bearing 4T1 breast tumors through intravenous injection. Because pH-responsive drug delivery systems have been explored before for cancer treatment, we have also prepared a typical pH-responsive nanocarrier.^57^ Interestingly, 48 h after the treatment, delivery of the drug via the lactate-responsive nanoparticles, but not free doxorubicin or delivery with pH-responsive particles, led to a marked reduction in tumor size and bioluminescence (Figures 4A and 4B). Kaplan-Meier survival analysis of animals receiving weekly injection of the drug indicates significantly enhanced survival when the lactate-responsive carrier was used (Figure 4C).

Tumor characteristics, such as tumor size, may affect the effectiveness of nanoparticle-mediated drug delivery.^59^^,^^60^ We examined therapeutic efficacy for TNBC at different sizes (starting tumor size at 130 or 385 mm^3^). Lactate-responsive delivery of doxorubicin leads to significantly enhanced inhibition of tumor growth in both groups, compared with free drug delivery (Figures S4A and S4B). The inhibition of tumor growth by nanoparticle-mediated delivery is lower for larger tumors. It is interesting that the delivery of empty nanoparticles also leads to a small but appreciable inhibition of TNBC growth (Figures S4A and S4C). It has been shown that hydrogen peroxide plays a multifaceted role in tumorigenesis.^61^^,^^62^ Although endogenous production of reactive oxygen species or hydrogen peroxide from cancer cells or tumor-associated matrix cells may promote tumorigenesis, a high level of hydrogen peroxide is cytotoxic and can inhibit tumor growth.^63^^,^^64^^,^^65^^,^^66^

Consistent with increased therapeutic effects, delivery of doxorubicin with the Janus particles led to significantly increased apoptosis and reduced cell proliferation in the tumor tissue, compared with free drug or delivery with pH-responsive particles (Figures 4D and S4C–S4E). Furthermore, the lactate-responsive nanoparticles were well tolerated in the animals. There was no significant change of body weight over 28 days with weekly injection of doxorubicin within the Janus particles (Figure S4F).

Ewing’s sarcoma is a type of rare tumor that mainly affects children and adolescents.^67^ Chemotherapy including doxorubicin remains the main modality of Ewing’s sarcoma treatment. To examine the potential efficacy in sarcoma treatment, we prepared tumor-bearing mice by subcutaneous injection of human SK-ES1 Ewing sarcoma cells into nude mice. Weekly injection of doxorubicin within the Janus nanoparticles showed greatly enhanced animal survival and significantly suppressed tumor growth *in vivo* (Figures 4E–4G). The median survival time increased to 19 days when the mice were treated with the Janus nanoparticles, compared to 10 days in control group, or 12 days in the cohort treated with free drug.

**Figure 5:**
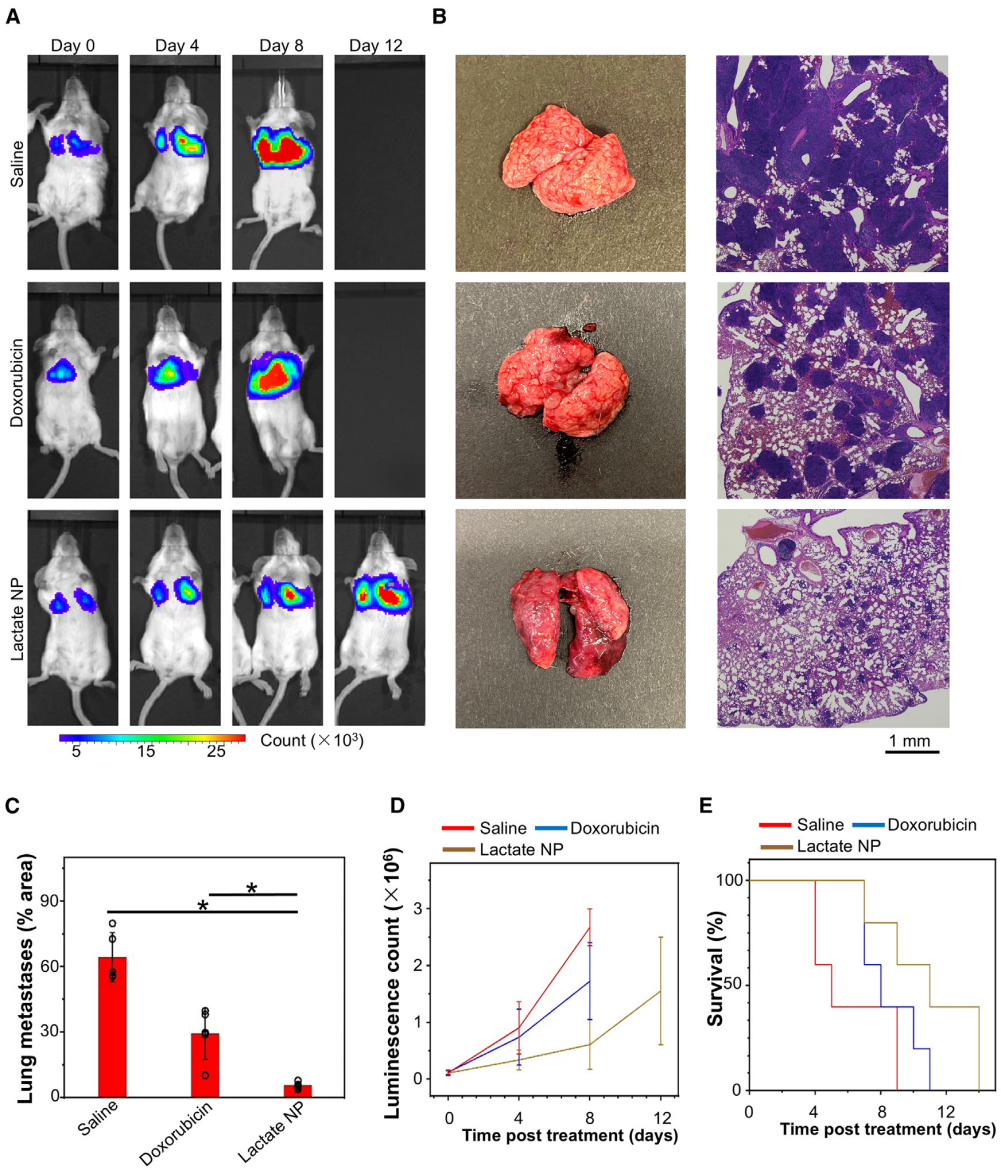
Lactate-responsive drug carriers increase the therapeutic efficacy for metastatic tumors (A) The bioluminescence images of lung metastasis (4T1 model) upon different treatments as indicated. (B) Lung images and hematoxylin and eosin (H&E) staining of the tissue 8 days after different treatments as indicated. (C) Quantification of lung metastasis with different treatments (percentage of tumor area in the lung from histology analysis). *n* = 5. Data are presented as mean ± SD. All error bars represent SD. ∗: *p* < 0.05 (Student’s t test). (D) Metastatic growth in the lung was monitored by bioluminescence imaging. *n* = 5. Data are presented as mean ± SD. All error bars represent SD. (E) Kaplan-Meier survival curve of mice with 4T1 lung metastasis model upon different treatments as indicated. *n* = 5.

Tumor metastasis is the most life-threatening aspect of cancer.^68^^,^^69^ We further evaluated the therapeutic potential of the lactate-responsive delivery system for the treatment of metastatic lesions using the lung metastasis model of breast cancers. Intravenous injection of *luciferase-*expressing 4T1 cells through tail vein led to rapid growth of lung metastases. Treatment with doxorubicin through the Janus particles markedly reduced the metastatic growth *in vivo*, as determined by bioluminescence imaging and histology analysis (Figures 5A–5D). Treatment using the Janus nanoparticles also significantly elongated animal survival (Figure 5E). Together, our data strongly suggest that the lactate-responsive drug delivery platform can greatly enhance the therapeutic efficacy of cancer chemotherapy for both primary tumors and metastatic diseases.

### Delivery of immunotherapeutic agents with the lactate-targeting Janus nanoparticles

**Figure 6:**
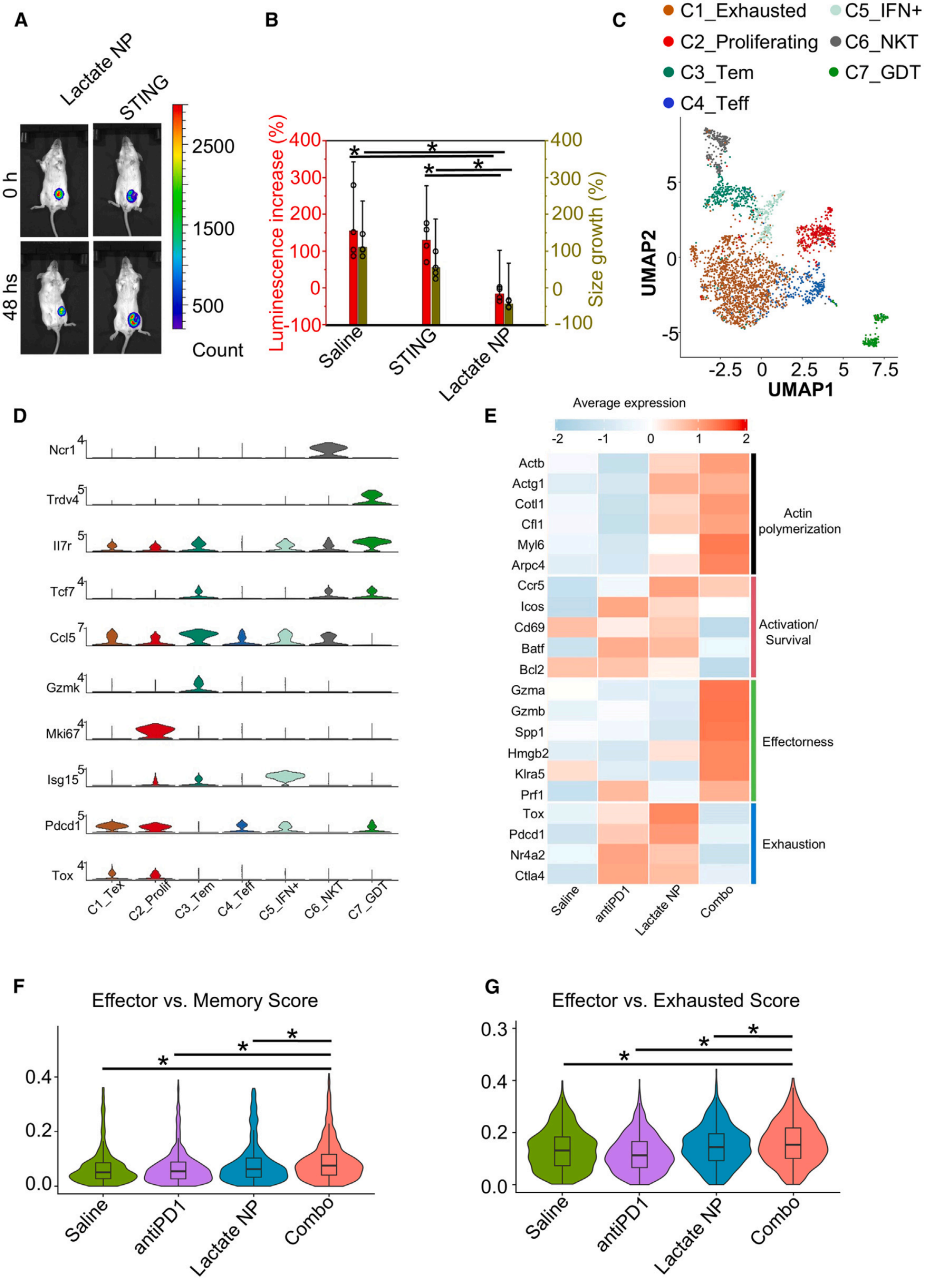
Delivery of immunotherapeutic agent with the lactate-responsive nanocarriers (A) Monitoring of growth of 4T1 tumors by bioluminescence imaging with different treatments as indicated. (B) Changes in bioluminescence signal and tumor size upon different treatments as indicated. *n* = 4. Data are presented as mean ± SD. All error bars represent SD. ∗: *p* < 0.05 (Student’s t test). (C) Uniform manifold approximation and projection (UMAP) embeddings of 2,931 total CD8^+^ T cells across four treatment conditions, colored by functional subset. Tex, T exhausted; Prolif, proliferating; Tem, T effector memory; Teff, T effector; NKT, natural killer T cell; GDT, γδ-T cell. (D) Violin plots of normalized gene expression of subset-specific markers. (E) Heatmap comparing functional gene expression across conditions in CD8^+^ T cells. Legend color denotes *Z*-scored expression. (F and G) Violin plots comparing effector gene module scores with memory gene module scores (F), and exhausted gene module scores (G) of different treatments in CD8^+^ T cells.

Cancer immunotherapy, such as immune checkpoint blockade (ICB), has demonstrated promising efficacy in the treatment of many human malignancies.^70^^,^^71^^,^^72^^,^^73^^,^^74^ Immune suppression of tumors entails different mechanisms, including suppressive cytokine secretion, lack of antigen presentation, apoptosis of T cells, and hostile metabolic states and nutrient deprivation in the tumor microenvironment.^75^^,^^76^ Thus, immune interventions can potentially reverse the suppressive immune microenvironment and enhance the efficacy of immunotherapy.^77^^,^^78^ Stimulator of interferon genes (STING) links innate immune responses to multiple downstream biological processes ranging from anti-tumor immunity to microbiome homeostasis.^79^ Activation of STING proteins induces the release of type I interferon and other proinflammatory cytokines. Pharmacological stimulation of STING pathway is a promising strategy for cancer immunotherapy.^80^ However, systematic stimulation of STING pathway is associated with strong immune-related toxicity and side effects.^79^^,^^81^ To examine the potential of STING activation by lactate-responsive targeting delivery, we tested loading of a non-nucleotide, small-molecule STING agonist, SR-717, into our Janus nanocarrier.^82^ Interestingly, delivery of SR-717 with the Janus particles to mice bearing 4T1 breast cancer leads to significant tumor shrinkage compared with control or free drug injection (Figures 6A and 6B), suggesting that the metabolic targeting of SR-717 can enhance the efficacy of cancer immunotherapy.

Although breast cancers were initially considered to have low immunogenic potential (cold tumor) with low mutational load, accumulating preclinical and clinical evidence demonstrated promising responses to ICB coupled with chemotherapies, suggesting that the tumor microenvironment of breast cancers can be therapeutically changed to enhance response to immunotherapy.^83^ To assess how the Janus nanoparticles can change the intratumoral immune landscape, we performed single-cell RNA sequencing (scRNA-seq) on tumor-infiltrating CD8^+^ T cells from 4T1 breast tumors upon STING agonist delivery with or without α-PD1 antibody treatment. Following quality control filtering, we recovered 2,931 cells to perform downstream analyses. Upon performing integration, dimensionality reduction, and clustering, we discovered 7 total clusters exhibiting distinct gene expression signatures (Figure 6C). The two largest clusters were annotated as exhausted (*C1_Exhausted*) and proliferating (*C2_Proliferating*) CD8^+^ T cells (Figures 6C and S5A)*.* Cells in the *C1_Exhausted* cluster highly expressed the inhibitory gene *Pdcd1* and the exhaustion-associated transcription factor *Tox*,^84^ while cells in the *C2_Proliferating* cluster highly expressed the proliferation marker *Mki67* (Figure 6D). We also found 1 effector memory cluster, annotated as *Ccl5+Il7r+Tcf7+Gzmk+* effector memory (*C3_Tem*), and 2 effector clusters, annotated as *Ccl5+Il7r-Tcf7-Gzmk-* effector (*C4_Teff*) and *Isg15+* interferon-high (*C5_IFN+*). Finally, we found 2 innate-like clusters, annotated as *Ncr1+* natural killer T (*C6_NKT*) and *Trdv4+* (*C7_GDT*) γδ cells. To examine treatment-specific transcriptomic differences, we carried out differential gene analysis across conditions (Figure 6E). Among all conditions, CD8^+^ T cells in the combo treatment group (STING agonist within the Janus particles + α-PD1 antibody) displayed the highest expression of genes related to effector function (such as *Gzma*, *Gzmb*, *Spp1*, and *Prf1*) and actin polymerization (such as *Actb*, *Actg1*, and *Cotl1*). Furthermore, with combo treatment, CD8^+^ T cells displayed the lowest expression of exhaustion-related genes (*Tox*, *Pdcd1*, *Nr4a2*, and *Ctla4*). In contrast, with STING agonist treatment alone or α-PD1 antibody treatment alone, CD8^+^ T cells both exhibited higher levels of activation markers *Cd69* and *Icos* as well as survival regulators *Bcl2* and *Batf* compared to the combo treatment, but both expressed lower levels of effector genes and higher levels of exhaustion genes. Gene module scoring further confirmed an upregulation of effector function in the combo treatment CD8^+^ T cells (Figures 6F and 6G). These results suggest that STING agonist delivery with the Janus nanoparticles can enhance the functionality of CD8^+^ T for ICB treatment of breast cancer.

**Figure 7:**
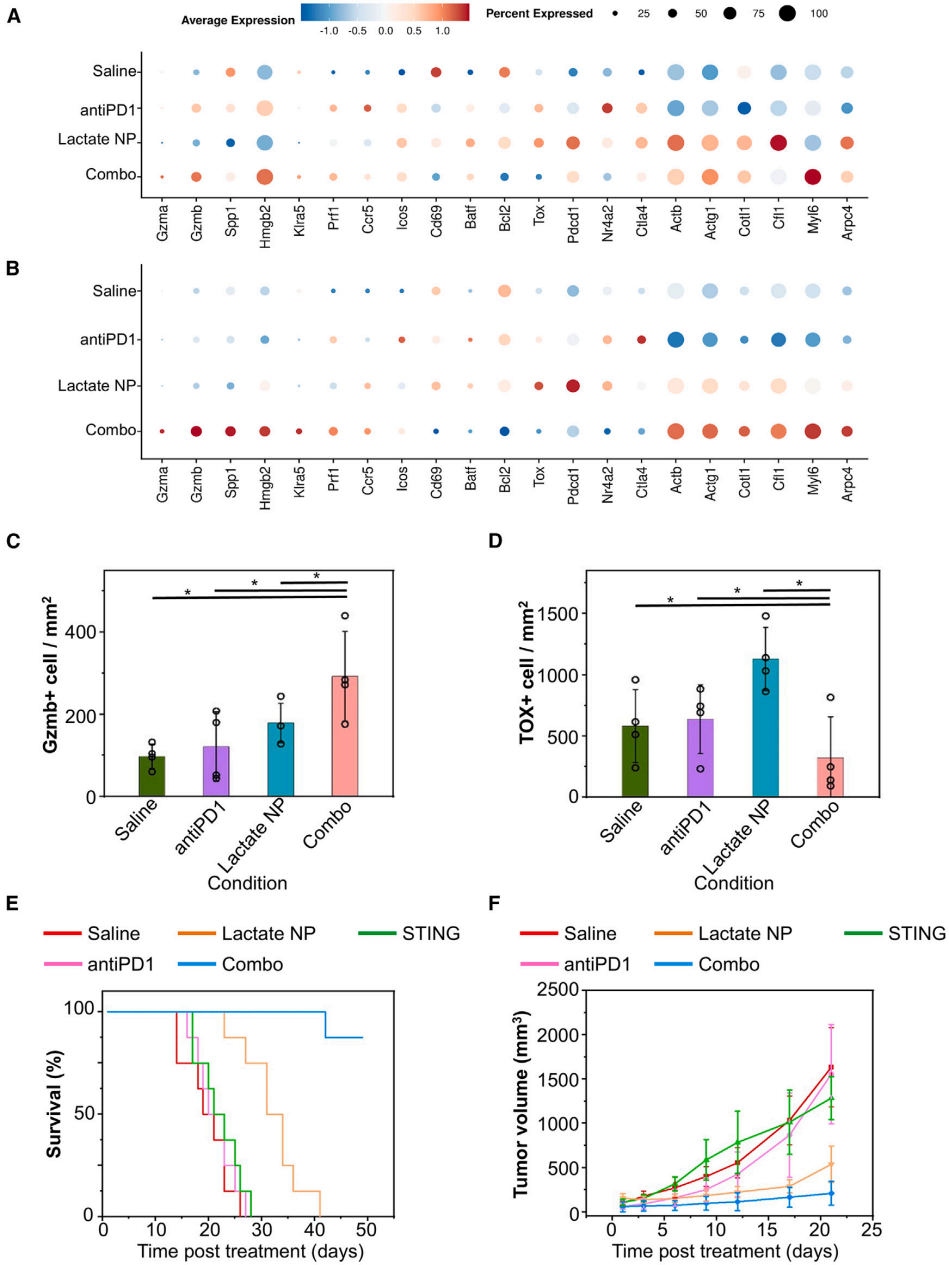
Delivery of STING agonist with the lactate-responsive nanocarrier can enhance the efficacy of immunotherapy for breast cancer (A and B) Dotplot comparing functional gene expression across conditions in proliferating (A) and exhausted (B) CD8^+^ T cells. (C and D) The tumor microenvironment in the combo treatment group presented significantly accumulated Gzmb+ cells and the lowest TOX level. Quantitation of the positive expression of the tumor-infiltrating granzyme B+ (C) and TOX+ (D) cells in different treatment groups. *n* = 4. Data are presented as mean ± SD. All error bars represent SD. ∗: *p* < 0.05 (Student’s t test). (E) Kaplan-Meier survival curve of mice with 4T1 lung metastasis model upon different treatments as indicated. *n* = 8. (F) Tumor growth was monitored by bioluminescence imaging upon different treatments as indicated. *n* = 8. Data are presented as mean ± SD. All error bars represent SD.

We next questioned which CD8^+^ T cell subsets are responding most significantly to combination treatment. By performing pairwise Augur analysis,^85^ we found *C2_Proliferating* and *C1_Exhausted* clusters to be highly ranked in distinguishing the combo-treated CD8^+^ T cells from the monotherapy groups (Figures S5B and S5C). Different treatments do not significantly affect the abundance/frequency of different T cell subpopulations (Figure S5D). In agreement with previous findings, in the combination treatment, the *C2_Proliferating* CD8^+^ T cells expressed the highest levels of effectorness-related genes and the lowest levels of exhaustion-related genes, although at modest levels (Figures 7A and S6A–S6F). CD8^+^ T cells isolated from tumors treated with STING agonist alone expressed significantly higher *Mki67* than the combo treatment CD8^+^ T cells, as well as higher levels of actin genes *Actb* and *Actg1* (Figures S6G–S6I). What’s more, we found that the combo treatment CD8^+^ T cells in the *C1_Exhausted* cluster have the highest expression of effector-related genes and actin-related genes, while expressing the lowest levels of exhaustion-related genes (Figures 7B and S6J–S6O). We found these differences to be more significant compared to those seen in the *C2_Proliferating* cluster. Altogether, our results suggest that the STING agonist delivery with the Janus nanoparticles can activate and expand CD8^+^ T cells, enhancing breast cancer immunotherapy when combined with ICB.

To validate scRNA-seq data acquired from CD8^+^ T cells, we performed immunohistochemical staining of the tumor in different treatment groups to assess the expression of genes associated with effectors and exhaustion. As shown in Figures 7C and S6P, compared to other treatment, Gzmb+ cells were significantly accumulated in the tumor in the combo treatment group, which was indicative of cytolytic function of the combination treatment, serving as a useful predictive biomarker for efficacious responses to immunotherapy.^86^^,^^87^^,^^88^^,^^89^ Thymocyte selection-associated high mobility group box protein (TOX) has been demonstrated as the key inducer of canonical features of T cell exhaustion and as an initiator of the exhausted T cell-specific epigenetic program. Figures 7D and S6P show that combination treatment group presented the lowest TOX level in the tumor microenvironment.^90^^,^^91^^,^^92^

To determine the therapeutic relevance of our findings, we carried out experiments to test the combination treatment with the 4T1 breast cancer model (Figures 7E and 7F). While direct injection of SR-717 shows only a marginal effect on tumor growth and animal survival, delivery of SR-717 within the Janus nanoparticles significantly inhibits tumor growth and improves animal survival. When combined with α-PD1 antibody treatment, the combination treatment demonstrates remarkable therapeutic efficacy with greatly reduced tumor size and elongated animal survival, compared to monotherapies. Seven out of the eight animals in the combination treatment cohort survived more than 50 days. No significant weight loss or histological changes in the major organs were found in any of the test groups, suggesting no severe side effect for STING agonist delivery with the Janus nanoparticles (Figure S7). Together, our results strongly suggest that the lactate-responsive nanocarrier provides a versatile and effective platform for enhancing therapeutic efficacy of different cancer therapies.

## Discussion

Metabolic reprogramming of tumor cells is a hallmark of cancer. Among all the changes, the metabolic adaption to aerobic glycolysis is believed to be critical for tumor cell proliferation and cancer progression.^14^^,^^15^^,^^16^^,^^17^ Thus, the Warburg effect is not only a hallmark of cancer but also a promising therapeutic target for treatment. Many enzymes involved in the aerobic glycolysis have been exploited for cancer therapies, such as glucose transporters, pyruvate kinase isozyme M2, and mammalian target of rapamycin kinase. However, due to the complexity of tumor metabolism, it is highly challenging to develop an effective treatment by targeting a single enzyme or a signaling pathway involved in the Warburg effect. In this study, we pursue a different strategy to leverage the cancer Warburg effect and develop a tumor-specific drug delivery system by targeting lactate itself. We developed and tested a lactate-oxidase-functionalized Janus nanoparticle system to deliver different chemotherapeutic or immunotherapeutic drugs for the treatment of primary and metastatic breast cancer and Ewing’s sarcoma. Our results demonstrate superior specificity of drug delivery with this platform, which led to greatly enhanced therapeutic efficacy *in vivo*. As the Warburg effect is one of the most common changes in tumor metabolism, our approach holds potential for treatment of many other solid tumors with accumulation of lactate.

Targeted delivery of cancer drugs can enhance therapeutic efficacy and reduce systemic toxicity and potential side effects. Aiming at improving the solubility and bioavailability of therapeutic molecules (e.g., small-molecule inhibitors, chemotherapy, RNAi, etc.), altering their bio-distribution, and facilitating their entry into the target cell, substantial progress has been made toward our understanding of how passive or active drug carriers interact with cells and tissues.^93^ In addition to conventional passive or active drug targeting, another attractive strategy is developing stimuli-responsive drug carriers that can respond to intrinsic or extrinsic signals, such as pH, enzymes, heat, ultrasound, or magnetic field to release drugs “on-demand” in a spatiotemporally controlled fashion.^1^ However, depending on the choice of stimulus and the sensitivity of the carriers, the development of stimuli-responsive strategies is hindered by tumor heterogeneity, insufficient specificity, limited tissue penetration of the stimuli, and poor spatial control of the stimulus.^94^^,^^95^^,^^96^^,^^97^ Our lactate-responsive drug carrier leverages both the EPR and Warburg effects of tumor, representing a more effective and specific approach for drug delivery by metabolic targeting of tumors.

The Janus particles are a unique class of asymmetry particles that combine two distinct chemical or physical functions on their opposite sides, which offers an opportunity to access multifunctional assembled structures that might not be possible to achieve with symmetric particles.^98^ They can be utilized to construct materials with controlled biological, chemical, and topographical heterogeneity. Chemical catalytic compounds or biocatalytic enzymes can be embedded or coated on either side of Janus particles, making them a versatile nanoplatform for drug delivery.^99^^,^^100^ Surfaces and interfaces of the Janus particles possessing distinct functions would be responsive to different stimuli, thus making it possible to alter their properties on demand. This unique asymmetric structure of Janus particles also allows them to be further constructed for dual-functional properties such as pH-near-infrared, computed tomography-magnetic resonance imaging, or pH-temperature dual response for synergistic cancer therapy.^101^^,^^102^^,^^103^^,^^104^ However, despite the numerous approaches for the fabrication of Janus particles in a laboratory setting, a significant barrier is the production scalability, which is critical for the application of Janus nanoparticles on an industrial scale. To achieve lactate-gated drug release, alternative strategy could be used to functionalize nanoparticles with lactate oxidase. Instead of anchoring the enzyme on the surface of the Au particles, we found that covalently crosslinking the enzyme with cyclodextrin (the capping molecules) could make nanocarriers with similar sensitivity toward lactate. Additionally, the H_2_O_2_-cleavable chemical links can also be utilized for the construction of H_2_O_2_-responsive hydrogel systems. When intercalated with lactate oxidase, these hydrogel systems may also possess the potential for the lactate-responsive drug delivery.

The increase of lactate is associated with other pathological conditions, such as arthritis, hypovolemia, septic shock, cardiac arrest, etc.^105^^,^^106^ Thus, the lactate-responsive drug carrier may have applications beyond cancer. For instance, chronic inflammation can lead to increased lactate levels in the synovial fluid of patients with arthritis. For patients with septic arthritis, the lactate level can reach ∼30 mM in the synovial fluid. A lactate-inducible delivery of anti-inflammatory drugs can achieve specific targeting of the disease and avoid systemic suppression of immunity. In sports medicine, extreme exercise can lead to rhabdomyolysis, which is also associated with an acute lactate elevation in muscle. The lactate-responsive drug delivery can be used for potential^107^^,^^108^ treatment of rhabdomyolysis, which can be fatal or result in permanent disability.^109^^,^^110^ However, potential elevated lactate level in noncancerous tissue also makes it essential to screen and stratify patients for lactate-responsive delivery of cancer therapeutics. Lactate level in tumor or other organs can be determined by metabolic imaging clinically, such as magnetic resonance spectroscopy imaging.^111^^,^^112^^,^^113^^,^^114^ Non-invasive imaging provides a robust approach to screen and enroll patients for future clinical tests of the drug delivery system, which can ensure safety and enhance tumor-specific drug delivery.

Immunotherapy is an emerging pillar for cancer treatment.^70^ Tumor immune microenvironment can be altered by chemotherapy, which can enhance efficacy of cancer immunotherapy.^115^^,^^116^^,^^117^ Notably, doxorubicin can induce immunogenic cell death in tumor cells, promoting the release of damage-associated molecular patterns, which, in turn, can facilitate the priming of tumor-specific CD8^+^ T cells and activate STING pathway.^118^ As a versatile drug delivery platform, it will be interesting to determine whether the Janus nanoparticles can co-deliver chemotherapeutic and immunotherapy drugs, such as doxorubicin and SR717 for cancer treatment.

In closing, engineering enzyme-functionalized Janus nanoparticles for lactate-inducible drug delivery represents a significant advancement toward metabolic drug targeting and cancer therapy, which can be also applied to other diseases with increased lactate levels.

### Limitations of the study

Limitations of the study include the following: first, the animal models for breast cancer or Ewing’s sarcoma may not fully recapitulate the heterogeneity and complexity of human cancers, necessitating further validation in more clinically relevant models. Additionally, while the platform leverages elevated lactate concentrations in tumors, lactate levels may vary between different tumor types or within different regions of the same tumor, potentially leading to inconsistent drug release. Future experiments should include stability and immunogenicity analysis of lactate oxidase *in vivo*, additional cancer models including patient-derived xenograft models, and detailed pharmacokinetic and biodistribution analyses to further evaluate targeted drug delivery.

## Resource availability

### Lead contact

Further information and requests for resources and reagents should be directed to and will be fulfilled by the lead contact, Xiaoyang Wu (xiaoyangwu@uchicago.edu).

### Materials availability

This study did not generate new unique reagents.

### Data and code availability

• scRNA-seq data have been deposited at GEO and are publicly available as of the date of publication. Accession numbers are listed in the key resources table. Microscopy data reported in this paper will be shared by the lead contact upon request.
• This paper does not report original code.
• Any additional information required to reanalyze the data reported in this paper is available from the lead contact upon request.

## Acknowledgments

We are very grateful to Dr. Bozhi Tian at the University of Chicago for sharing reagents and technical assistance. We thank Linda Degenstein at the transgenic core facility at the University of Chicago for excellent technical assistance. The animal studies were carried out in the ALAAC-accredited animal research facility at the University of Chicago. This work was supported by 10.13039/100000002NIH grants R01OD023700, R21AR080761, R01DA047785, and R01AR78555, 10.13039/100000884Cancer Research Institute (CRI) Technology Impact Award, 10.13039/100001384Samuel Waxman Cancer Research Foundation, 10.13039/100012640Alan B. Slifka Foundation and Israel Cancer Fund for Pediatric Sarcoma Grant, 10.13039/100003287Rally Foundation Outside the Box Grant, University of Chicago Comprehensive Cancer Center (UCCCC) Duckworth Family Commercial Promise Award, Cancer Immunotherapy Team Science Award, Pancreatic Cancer SPORE grant, UCHAP pilot award, and Ullman Family Team Science Award (to X.W.) and 1DP2AI144245 (to J.H.).

## Author contributions

X.W., J.Z., and J.H. designed the experiments. J.Z., J.L., S.A.K., S.G., E.T., Y.H., L.C., and A.H. performed the experiments. J.Z., T.P., and X.W. analyzed the data. X.W., J.H., and J.Z. wrote the manuscript. All authors edited the manuscript.

## Declaration of interests

J.Z. and X.W. are inventors for patent PCT/US2020/070052 (Lactate response system and methods). X.W. is a co-founder of Alnair Therapeutics, Inc.

## STAR★Methods

### Key resources table

**Table:** 

REAGENT or RESOURCE	SOURCE	IDENTIFIER
**Antibodies**		

Ultra-LEAF™ Purified anti-mouse CD279 (PD-1) Antibody	BioLegend	Cat#135250; RRID: AB_2783093
Recombinant Anti-Granzyme B antibody [EPR22645-206]	Abcam	Cat#ab255598
Anti-TOX antibody	Abcam	Cat#ab155768

**Chemicals, peptides, and recombinant proteins**		

Aminopropyltriethoxysilane (APTES)	Sigma-Aldrich	A3648; CAS: 919-30-2
Dimethyl Sulfoxide (DMSO)	Sigma-Aldrich	276855; CAS: 67-68-5
((3-Mercaptopropyl)trimethoxysilane	Sigma-Aldrich	175617; CAS: 4420-74-0
N-(3-dimethylaminopropyl)-N′-ethylcarbodiimide hydrochloride (EDC)	Sigma-Aldrich	03450; CAS: 25952-53-8
N-hydroxysuccinimide (NHS)	Thermo Fisher Scientific	AC157270250; CAS 6066-82-6
4-(carboxymethyl)phenylboronic acid pinacol ester (CAPE)	Sigma-Aldrich	718831; CAS: 797755-07-8
Cetyltrimethylammonium bromide (CTAB)	Sigma-Aldrich	H9151; CAS: 57-09-0
Ethanol	Sigma-Aldrich	459836; CAS: 64-17-5
Paraffin wax (mp 53°C–58°C)	Sigma-Aldrich	327204; CAS: 8002-74-2
Hydrogen tetrachloroaurate(III) (HAuCl4·3H2O)	Sigma-Aldrich	520918; CAS: 16961-25-4
Sodium citrate tribasic dihydrate	Sigma-Aldrich	S4641; CAS: 6132-04-3
Doxorubicin hydrochloride (DOX)	Sigma-Aldrich	D1515; CAS: 25316-40-9
*o*-Phenylenediamine	Sigma-Aldrich	P23938; CAS: 95-54-5
3-Mercaptopropionic acid	Sigma-Aldrich	M5801; CAS: 107-96-0
Tris(bipyridine)ruthenium(II) chloride	Sigma-Aldrich	224758; CAS: 50525-27-4
DL-Lactic Acid Lithium Salt	Thermo Fisher Scientific	ICN10082405; CAS: 867-55-0
4-Aminoantipyrine	Sigma-Aldrich	06800; CAS: 83-07-8
3-(N-Ethyl-3-methylanilino)-2-hydroxypropanesulfonic acid sodium salt	Sigma-Aldrich	E8631; CAS: 82692-93-1
Sodium cholate	Thermo Fisher Scientific	18-606-437; CAS: 361-09-1
Hydrogen peroxide solution	Sigma-Aldrich	HX0640; CAS: 7722-84-1
3-(trimethylsilyl)-2,2′,3,3′- tetradeuteropropionic acid	Sigma-Aldrich	269913; CAS: 24493-21-8
α-Cyclodextrin (α-CD)	Sigma-Aldrich	C4642; CAS: 10016-20-3
Cisplatin	Sigma-Aldrich	PHR1624; CAS: 15663-27-1
Oxaliplatin	Sigma-Aldrich	PHR1528; CAS: 61825-94-3
Cell proliferation reagent WST-1	Sigma-Aldrich	CELLPRO-RO
Chitosan	Sigma-Aldrich	417963; CAS: 9012-76-4
Alginate	Sigma-Aldrich	PHR1471; CAS: 9005-38-3
3,3′-Diethylthiadicarbocyanine iodide	Sigma-Aldrich	173754; CAS: 514-73-8
Bis[3,4,6-trichloro-2-(pentyloxycarbonyl) phenyl] oxalate	Thermo Fisher Scientific	O02365G; CAS: 30431-54-0
Bovine serum albumin	Sigma-Aldrich	A2153; CAS: 9048-46-8
Pluronic F-127	Sigma-Aldrich	P2443; CAS: 9003-11-6
Lactate oxidase	Thermo Fisher Scientific/Toyobo U.S.A.	NC1207312
Mesoporous silica nanospheres (MCM-41 type, 3 ± 1 nm pore diameter)	nanoComposix	SHSD100
SR-717	MedChemExpress U.S.A.	Cat#HY-131454; CAS: 2375421-09-1

**Critical commercial assays**		

Chromium Next GEM Single Cell 5′ Reagent Kits v2	10x Genomics	1000263
Qubit dsDNA HS Assay Kit	Invitrogen	Q32851
5′ Feature Barcode Kit	10x Genomics	1000256
Lactate Assay Kit	Sigma	MAK064

**Deposited data**		

Single-cell RNA sequencing	This paper	GEO: GSE226922; Accession number: avqnkeumdradzwd

**Experimental models: Cell lines**		

Mouse: 4T1-luc2 cells	ATCC	CRL-2539-LUC2
Human: SKES1 cells	ATCC	HTB-86

**Experimental models: Organisms/strains**		

Female BALB/c mouse	Jackson laboratory	Stock 000651
Female athymic nude mouse	Jackson laboratory	Stock 007850
WT CD1 mouse	Transgenic Core Facility/University of Chicago	N/A

**Software and algorithms**		

ImageJ	NIH	https://imagej.nih.gov/ij/
Excel	Microsoft	https://products.office.com/en-us/excel
OriginLab	OriginLab	http://www.originlab.com/
FlowJo	Becton, Dickinson & Company	https://www.flowjo.com/
GraphPad Prism	Dotmatics	https://www.graphpad.com/

### Experimental model and study participant details

#### Cell lines and cell culture

American Tissue Culture Collection (ATCC) provided the mouse 4T1-luc2 cell line and human SKES1 cell line. 4T1-Luc2 cells are a luciferase-expressing cell line derived from parental line CRL-2539 by transduction with lentiviral vector encoding firefly luciferase gene (luc2) under control of EF-1 alpha promoter. SK-ES-1 is a cell line exhibiting epithelial morphology that was isolated from the bones of an 18-year-old, White male patient with anaplastic osteosarcoma. 4T1-luc2 and SK-ES-1 cell line were grown in Dulbecco’s Modified Eagle Medium (DMEM) (supplemented with 10% fetal bovine serum, 100 U/mL penicillin, and 100 U/mL streptomycin). During cell culture, cells were kept in an incubator at 37°C with 5% CO2. The cell lines were routinely tested to confirm the absence of mycoplasma.

#### Model animal studies

Female BALB/c and athymic nude mice (10–12 weeks old) were purchased from Jackson laboratory. WT CD1 (female, 10–12 weeks old) mice were obtained from the Transgenic Core Facility at University of Chicago. All mice used in this study were bred and maintained at the ARC (animal resource center) of the University of Chicago in accordance with institutional guidelines. All the experimental procedures on live animals were carried out in line with the Institutional Animal Care and Use Committee (IACUC) approved protocols (ACUP #: 72219) of the Animal Care Center at the University of Chicago. All the mice were housed under pathogen-free conditions in the ARC (Animal Resources Center) at the University of Chicago under a 12 h light-dark cycle. Housing facility maintains a temperature at 70–73° (average 72) and humidity at 40–50% (average 44%). Before injection, mice were anesthetized using continuous inhalation of isoflurane and had their injection site sterilized with ethanol. All the subjects were not involved in any previous procedures.

### Method details

#### Janus nanoparticles preparation

The Janus nanoparticles were prepared according to procedures inspired from the literatures and as described previously.^39^^,^^119^^,^^120^ First, citrate-capped Au nanoparticles with a diameter of ca. 20 nm were prepared based on the Turkevich–Frens method, by reduction of Au(III) using sodium citrate.^41^ After boiling 100 mL of 3 μM HAuCl_4_·3H_2_O, 1.5 mL of 1% sodium citrate solution was added and stirred at 1000 rpm for 15 min (Lab centrifuge; Sorvall Legend Micro 21). The synthetized Au nanoparticles solution was cooled down to room temperature and a red solution was obtained. Second, 200 mg of mesoporous silica nanoparticles were added to an aqueous solution (6.7% ethanol) of CTAB (9 mL, 1 μM) and heated to 75°C. 1 g of wax is then deposited at the top of the particles suspension. After the wax melting, the mixture is submitted to vigorously mixing using a homogenizer for 15 min, operating at 9000 rpm. The mixture was further stirred for 1 h at 1500 rpm 75°C and cooled down to room temperature. Then, it was treated with 200 μL of (3-Mercaptopropyl)trimethoxysilane and 10 mL methanol for 3 h. The partially mercapto-functionalized mesoporous silica nanoparticles were isolated by centrifugation and washed with methanol. At last, to assemble the Janus particles, the nanoparticles were dispersed in 100 mL of methanol and added 400 mL of the as-synthesized Au nanoparticles solution. After stirring overnight, the Janus Au/silica nanoparticles were washed with ethanol and chloroform. Finally, the Janus Au/silica nanoparticles were dried under vacuum at room temperature. The morphology and size of the nanoparticles was characterized by transmission electron microscopy (TEM) (Tecnai Spirit TEM, working at 120 kV) and dynamic/electrophoretic light scattering (DLS) (Wyatt Möbiuζ).

#### Janus nanoparticles with mesoporous silica face functionalized by arylboronate

100 mg Au/silica nanoparticles was treated with 10 mL APTES solution (the volume ratio of APTES: ethanol: H_2_O is 15:950:35) overnight. Then, the nanoparticles with amine group functionalized mesoporous silica face was isolated by centrifugation and washed with ethanol and water thrice. The Janus Au/MS-NH_2_ nanoparticles were dried under vacuum at room temperature.

In order to prepare Janus nanoparticles with mesoporous silica face functionalized by arylboronate, 0.06 g CAPE, 0.025 g NHS and 0.05 g EDC was mixed in 2.5 mL DMSO for 15 min. Then, the as synthesized amine group functionalized nanoparticles in 2.5mL DMSO was added into the mixture. After stirring overnight, the Janus nanoparticles with mesoporous silica face functionalized by arylboronate were washed with DMSO and water. The Janus nanoparticles were dried under vacuum at room temperature.

Loading and capping of the Janus nanoparticles with mesoporous silica face functionalized by arylboronate

To prepare the Janus nanoparticles with mesoporous silica face functionalized by arylboronate nanoparticles loaded with doxorubicin, Oxaliplatin, Cisplatin, [Ru(bpy)_3_]Cl_2_, or SR-717 in the pores of mesoporous silica, 100 mg of Janus nanoparticles with mesoporous silica face functionalized by arylboronate was first dispersed in 10 mL of doxorubicin water solution containing 30 mg doxorubicin (or 10 mL of Oxaliplatin DMSO solution containing 30 mg Oxaliplatin; or 10 mL of Cisplatin DMSO solution containing 20 mg Cisplatin; or 10 mL of acetonitrile containing 25 mg of Tris(bipyridine)ruthenium(II) chloride, or 10 mL of SR-717 DMSO solution containing 30 mg SR 717) and further stirred for 24 h at 4°C in dark room. The resulting solid was filtered off, washed twice with water (or DMSO for Cisplatin, Oxaliplatin, and SR717 loaded particles) and dried under vacuum at 4°C.

To prepare the capped nanoparticles, the loaded Janus nanoparticles (100 mg) were dispersed in 2.5 mL of 0.1 M sodium phosphate buffer (PBS), with pH at 7.5, at 4°C. Then 250 mg of α-cyclodextrin in 2.5 mL of 0.1 M PBS was added, following by stirring for 24 h at 4°C in dark room. The resulting solid was filtered off, washed thrice with PBS and dried under vacuum at 4°C. The unloaded nanoparticles with α-cyclodextrin cap were also prepared using the same approach. Fourier transform infrared (FT-IR) analysis (Nicolet iS50 FTIR Spectrometer) and thermogravimetric analyzer (TA Instruments Discovery TGA) were employed for characterization of Janus nanoparticles.

#### Immobilization of lactate oxidase on the Au face of the janus nanoparticles

100 mg nanoparticles were first dispersed in 10 mL acetonitrile and 100 μL of anhydrous 3-mercaptopropionic acid was added. The suspension was stirred for 1 h. The resulting solid was filtered off, washed thrice with acetonitrile and re-suspended in 9 mL PBS. After that, 35 mg of EDC and 58.5 mg of NHS were added. The mixture was stirred at 4°C for 30 min 10 mg of lactate oxidase was then dispersed in 1 mL of cold PBS and added to the mixture. After stirring for 24 h at 4°C in dark room, the resulting Janus nanoparticles with lactate oxidase on the Au face was filtered off, washed thrice with cold PBS, freeze-dried (Freeze dryer; Virtis Benchtop; SP Industries, Inc.) and stored at 4°C.

#### Lactate oxidase anchored with 3A-amino-3A-deoxy-(2AS,3AS)-alpha-cyclodextrin

Dissolve 50 mg of N-(3-Dimethylaminopropyl)-N′-ethylcarbodiimide hydrochloride, 50 mg of N-Hydroxysuccinimide, and 85 mg 3A-Amino-3A-deoxy-(2AS,3AS)-alpha-cyclodextrin hydrate in 2 mL PBS buffer. Rotated for 1 h at 4°C. Add 10 mg of lactate oxidase into the solution. Rotated for 48 h at 4°C.Dialysis in DI-water at 4°C for 48 h with a 2 kDa dialysis tubing.

#### Mesoporous silica nanoparticles surface functionalized with arylboronate

500 mg of mesoporous silica nanospheres (MCM-41 type, 3 ± 1 nm pore diameter, 120 nm diameter), 0.75 mL of (3-Aminopropyl)triethoxysilane, 1.75 mL of DI-water and 30 mL of ethanol was mixed and vigorously ultrasonicated for 20 min. Add 17.5 mL of ethanol into the suspension. Stirred at room temperature for 16 h. Centrifuge down the particles and wash with ethanol for 3 times. Dry under vacuum. Disperse the particles in 25 mL of DMSO with ultrasonication. 0.29 g of 4-(carboxymethyl)phenylboronic acid pinacol ester, 0.125 g of N-Hydroxysuccinimide, and 0.25 g of N-(3-Dimethylaminopropyl)-N′-ethylcarbodiimide hydrochloride were dissolved in 12.5 mL of DMSO and stirred at room temperature for 15 min. Mix the solution with the particle suspension. Rotate the mixture at room temperature for 24 h. Centrifuge down the particles and wash with DMSO for 3 times and ethanol once. Dry under vacuum.

#### Doxorubicin loading and α-cyclodextrin capping of the lactate-responsive core/shell nanoparticles

150 mg of functionalized mesoporous silica nanospheres were dispersed in 3 mL of DI-water with ultrasonication. 15 mg of Doxorubicin was dissolved in 100 μl of DMSO and added into the suspension. Rotate the mixture at 4°C for 24 h in dark room. The above dialyzed lactate oxidase anchored with 3A-Amino-3A-deoxy-(2AS,3AS)-alpha-cyclodextrin solution was added into the suspension and rotated at 4°C for 16 h in dark room. 400 mg of α-cyclodextrin was added into the suspension and further rotated at 4°C for 16 h in dark room. Centrifuge down the particles and wash with DI-water for 3 times. Dialysis in DI-water at 4°C for 4 days with a 10 kDa dialysis tubing in dark room to remove attached Doxorubicin. Freeze-dry to get the finial lactate responsive particles loaded with Doxorubicin and stored at 4°C.

#### Enzymatic assay

The method from Toyobo is based on the following set of 2 reactions. One unit of lactate oxidase causes the formation of one micromole of hydrogen peroxide (half a micromole of quinoneimine dye) per minute at pH 7.4 at room temperature. The appearance of quinoneimine dye is measured at 555nm by spectrophotometry (Microplate reader; BioTek Synergy *neo*).

1. With lactate oxidase: L-Lactate + O₂ → Pyruvate + H₂O₂
2. With peroxidase: 2H₂O₂ + 4-Aminoantipyrine + N-ethyl-N-(2-hydroxy-3-sulfopropyl)-m-toluidine → Quinoneimine dye + 4H₂O

Pipette 1.0mL of working solution (8.0mL DL-Lactate solution (0.125M), 1.2mL 4-aminoantipyrine solution (0.5%), 0.8mL N-ethyl-N-(2-hydroxy-3-sulfopropyl)-m-toluidine solution (20 mM), 2.0mL Peroxidase solution (25 U/ml), 8.0mL distilled water) into a test tube and equilibrate at 37°C for about 5 min. Then add 0.05mL of the enzyme solution (Dissolve the enzyme preparation in ice-cold 20mM N--2-aminoethanesulfonic acid/Sodium Hydroxide pH7.0 containing 1mM Ethylenediaminetetraacetic acid and 0.5% (w/v) sodium cholate, and dilute to 0.04–0.1 U/ml with the enzyme diluent (20mM K-phosphate buffer, pH7.0 containing 0.1% (w/v) sodium cholate)) and mix. After 15 min at 37°C, add 2.0mL of Dodecyl sodium sulfate solution (0.25%) to stop the reaction and measure the optical density at 555nm against water (optical density test). At the same time, prepare the blank by using the same method as the test except that the enzyme diluent is used instead of the enzyme solution (optical density test blank). Activity can be calculated by using the following formula:

Weightactivity(U/mg)=(ODtest−ODblank)×Vt×df/(34.3×1/2×t×1.0×Vs×C)=ΔOD×0.237×df/C

df: Dilution factor.

Vt: Total volume (3.05mL).

Vs.: Sample volume (0.05mL).

34.3: Millimolar extinction coefficient of quinoneimine dye under the assay condition (cm^2^/micromole).

1/2: Factor based on the fact that one mole of H₂O₂ produced half a mole of quinoneimine dye

t: Reaction time (15 min).

1.0: Light path length (cm).

C: Enzyme (particles) concentration in dissolution (C mg/mL).

#### *In vitro* drug release assay

For *in vitro* drug release, 5 mg/mL drug loaded particle ensemble was dispersed in different solutions. The suspension was shaken at 200 rpm and 37°C. At predetermined time intervals, the suspension was centrifuged at 21000 rpm for 2 min and an aliquot (0.3 mL) was withdrawn. An equal volume of fresh medium was added to keep the volume constant.

The amount of released doxorubicin and tris(bipyridine)ruthenium(II) chloride was analyzed by microplate reader. Measurement of the fluorescence intensity of doxorubicin (doxorubicin: λex = 485 nm and λem = 590 nm; Tris(bipyridine)ruthenium(II) chloride: λex = 453 nm and λem = 595 nm) was used to determine the amount of doxorubicin and tris(bipyridine)ruthenium(II) chloride in the supernatants. The concentration of doxorubicin and tris(bipyridine)ruthenium(II) chloride was calculated by the standard curve and fluorescence intensity.

The withdrawn samples for Oxaliplatin and Cisplatin were analyzed by the *o*-phenylenediamine colorimetric assay. Samples (0.3 mL) were heated to 100°C, and added to 0.3 mL of *o*-phenylenediamine (2 mg/mL DMF) and kept at 100°C for 15 min. The amount of platinum in the sample was determined by measuring the UV−vis absorbance at 703 nm using cisplatin as a standard curve.

#### Breast cancer mouse model

To generate spontaneous breast cancer, 106 murine breast cancer 4T1-luc cells in 100 μL PBS were injected into the mammary fat pad in 8-week-old female BALB/c or nude mice under anesthesia with isoflurane 2%.

To generate breast cancer lung metastasis model, 2.5×10^5^ 4T1-luc cells in 100 μL PBS were intravenously injected into 8-week-old female BALB/c.^121^ The lung metastasis of 4T1-luc breast cancer was confirmed by IVIS after the IP injection of D-luciferin potassium.

Tumors were morphometrically evaluated daily with an electronic caliper and tumor volume was estimated using the formula: tumor volume = length x width^2^/2. Tumor and body weight measurements were performed using calipers and weigh scale respectively.

#### Ewing’s sarcoma mouse models

Athymic nude mice were implanted with subcutaneous flank tumors. At 6 to 8 weeks of age, the mice were injected with 0.2 mL of SKES1 cells suspended at a concentration of 1 × 10^7^ cells/0.1 mL into the right flanks of mice.^122^

#### Lactate concentration measurement

Lactate Assay Kit (Sigma, MAK064) was used for the lactate measurement. Samples of mouse tissue (BALB/c, in the range of 50 mg) were homogenized (Ultra-Turrax T-25 homogenizer; IKA), still frozen, with a biomasher (DiagnoCine) in 0.5 mL of chilled acetone: kit buffer, to a final proportion (including the expected water content of the samples) of 1.25:1. The homogenate was centrifuged; all proteins removed with the precipitate, and floating lipids, not soluble in the diluted acetone, were discarded. The acetone tissue extracts were used for the estimation of lactate.^123^

#### Hydrogen peroxide detection *in vivo*

The nanoreactor formulation for hydrogen peroxide detection according to procedures inspired from Dr. Sehoon Kim’s literature with slight modification.^124^ Briefly, 10 mg of Pluronic F-127, 50 mg of bovine serum albumin, 100 mg of bis[3,4,6-trichloro-2-(pentyloxycarbonyl) phenyl] oxalate and 2 mg of 3,3′-diethylthiadicarbocyanine iodide were homogeneously dispersed in 5 mL Milli-Q water and then vigorously shaken to afford an aqueous dispersion of nanoreactor formulation.

14 days after 4T1 breast tumor implantation, nude mice were used in this experiment. 1 mL of the as prepared nanoreactor formulation was intraperitoneally injected into nude mouse. After that, a vary amount of Janus nanoparticles lactate oxidase anchored Au/mesoporous silica was intravenously injected into mice through tail vein. All *in vivo* images were taken 1 min after Janus nanoparticles injection and imaging with 2 min acquisition (IVIS-200 imaging system; Xenogen).

#### Preparation of pH-responsive nanocarrier loaded with doxorubicin

The nanocarrier for pH-responsive delivery was prepared and loading content of doxorubicin in the nanocarrier was estimated as described in previous literature.^57^ At first, the surface of mesoporous silica nanoparticles was functionalized with amine groups by treatment with APTES using the same method as described above for Au/mesoporous silica nanoparticles. Alginate was dissolved in 0.5 M NaCl solution (pH was adjusted to 3.0 with acetic acid). After that, 25 mL of 2 mg/mL alginate in 0.5 M NaCl solution was added to 25 mL of 2.0 wt % aminated mesoporous silica suspension and stirred at room temperature for 2 h. The particles were centrifuged at 8000 rpm for 10 min, washed, and suspended with 0.5 M NaCl solution. Consequently, 25 mL of 2 mg/mL chitosan solution was added to the suspension and stirred for 2 h, followed by centrifugation, washing, and redispersing in 0.5 M NaCl solution. pH-responsive nanocarrier could thus be obtained by repeating the above operations alternately and consecutively twice. A total of 0.2 g of as prepared nanocarrier was added to 20 mL of 2 mg/mL doxorubicin solution in PBS at 25°C. The suspension was stirred for 12 h under dark conditions, followed by vacuum at room temperature for 2 h. The doxorubicin-loaded pH-responsive nanocarrier (pH NP) was collected by centrifugation at 8000 rpm for 10 min and washing with distilled water thrice. The loading content of doxorubicin loaded in the pH-responsive nanoparticles was estimated spectrophotometrically as 9.7%.

Loadingcontent(%)=100%∗(WeightofdoxorubicininpHnanoparticles)/(WeightofpHnanoparticles)

#### Biodistribution of doxorubicin *in vivo*

Liquid chromatography/mass spectrometry analysis for doxorubicin bio-distribution *in vivo* was performed according to procedures inspired from the literatures.^54^^,^^55^^,^^56^^,^^57^ For quantitative analysis, 52 BALB/c mice, 14 days after 4T1 breast tumor implantation, were assigned into four groups, including saline control (12 mice), free doxorubicin (12 mice), lactate-responsive nanoparticles (16 mice), and pH-responsive nanoparticles (12 mice) groups. The mice in each experimental group were intravenously injected with 500 μL of sterile saline suspension of free doxorubicin, Lactate nanoparticles, or pH nanoparticles at the dose of 4 mg doxorubicin/kg. Injections of sterile saline at equivalent volume were given to mice as control. 3–4 mice in each group were sacrificed at 0.5, 1, 2, and 4 h after drug administration. Immediately after sacrifice, the tumor, liver, kidneys, thigh muscle, and heart were excised from each mouse and washed with distilled water to remove the surface blood and impurities. Blood was obtained by cardiac puncture. Around 30 mg of tissue was frozen in liquid nitrogen and freeze fractured using a liquid nitrogen cooled bio-masher. A total of 400 μL of a 50 mM ascorbic acid buffer with 2 mM D-L saccharic acid raised to a pH of 4.5 by titration with 1 M NaOH was added to the tissue powder. This buffer solution stopped any enzymatic degradation of doxorubicin within the tissue. The 400 μL homogenate samples were extracted using a chloroform–methanol (4 : 1) by mixing for 3 min and centrifuging for 15 min at 10 000 rpm. The upper organic layer was transferred into a new tube and evaporated to dry under a stream of nitrogen. The dried residue was reconstituted with 200 μL of methanol. A 10 μL aliquot of the supernatant was injected into the liquid chromatography/mass spectrometry. For the standard curve, calibration standard samples with known concentrations of doxorubicin were prepared in tissue extracts from blank mice (without drug administration) by adding different volumes of the freshly prepared doxorubicin solution into the extracts.

An Agilent 1290 LC system coupled with an Agilent 6540 UHD Q-TOF was used to perform the liquid chromatography/mass spectrometry analysis by using positive ion mode electrospray ionization (ESI) as the ion source with source voltage of 3.5 kV, sheath gas temperature of 350°. A ZORBAX Eclipse Plus C18 column (ID 2.1 mm length 100 mm, particle size 1.8 μm) with guard column was employed for LC separation by using water with 0.1% formic acid as the mobile phase A and pure methanol as the mobile phase B. The LC flow rate was set at 0.20 mL/min with 40% phase A and 60% phase B. Extracted ion chromatogram is created by plotting the intensity of the signal observed at chosen mass-to-charge value for doxorubicin at m/z 544.1815. The peak area ratio related to the calibration standard curve of standard samples was used for the quantification of doxorubicin.

Ex *vivo* imaging was also performed according to procedures inspired from the literatures.^125^^,^^126^^,^^127^^,^^128^^,^^129^ The mice were sacrificed 1 h after drug administration, and tissues (tumor, heart, liver, thigh muscle and kidney) were extracted, washed with PBS. The images were captured immediately by IVIS. The fluorescence channel was set as 465/580 nm excitation/emission.

#### Therapeutic efficacy in breast cancer mouse models

For analyzing short term therapeutic efficacy with doxorubicin loading, 20 BALB/c mice, 14 days after 4T1 breast tumor implantation, were assigned into five groups, including saline control, free doxorubicin, pH-responsive nanoparticles, and lactate-responsive nanoparticles groups. 4 mice in each experimental group were intravenously injected with 200 μL of sterile saline suspension of free doxorubicin, pH-responsive nanoparticles, and lactate-responsive nanoparticles at the dose of 4 mg doxorubicin/kg. Injections of sterile saline at equivalent volume were given to mice as control. Body weight, tumor size and luminescence measurements were performed 48 h after injection.

In order to perform the histological analysis, all mice were sacrificed and the tumors were excised, fixed in formalin, embedded in paraffin, and sectioned. Hematoxylin and eosin (H&E), Ki67, Caspase 3 staining was used for histological observations. All specimen evaluation was performed on an Olympus microscope using an ocular magnification of×2 and ×60. Ten to 20 fields per tumor were examined depending on its cellularity (minimum 400 tumor cells). The ratios of proliferation or apoptosis were obtained by calculating the percentage of Ki67 or caspase3-positive cells and comparing with the total number of cells in the same areas. Each datum was counted in a minimum of four randomly selected areas in immunohistochemistry staining images. Control slides (minus primary antibody) were assessed for non-specific binding before assessing the percentage of tumor cell binding the Ki67 and cleaved Caspase 3 antibodies. Areas of normal and benign breast were excluded from the final assessment.

For analyzing long term therapeutic efficacy with doxorubicin loading, 40 BALB/c mice, 14 days after 4T1 breast tumor implantation, were assigned into five groups, including saline control, free doxorubicin, pH-responsive nanoparticles, and lactate-responsive nanoparticles groups. 8 mice in each experimental group were intravenously injected once per week with 200 μL of sterile saline suspension of free doxorubicin, pH-responsive nanoparticles, and lactate-responsive nanoparticles at the dose of 4 mg doxorubicin/kg. Injections of sterile saline at equivalent volume were given to mice once per week as control. Mice were euthanized once the tumor reaches 1500 mm^3^. Tumor volumes were recorded after treatment on day 1, 3, 6, 9, 12, 17, and 21.

For analyzing therapeutic efficacy for lung metastasis, 15 BALB/c mice with 4T1 breast cancer lung metastasis, 6 days after 4T1 breast tumor cell iv injection, were assigned into 3 groups, including saline control, free doxorubicin, and doxorubicin loaded lactate-responsive nanoparticles groups. 5 mice in each experimental group were intravenously injected once every 4 days with 200 μL of sterile saline suspension of free doxorubicin, and doxorubicin loaded lactate-responsive nanoparticles at the dose of 4 mg doxorubicin/kg. Injections of sterile saline at equivalent volume were given to mice once every 4 days as control. The lung metastasis of 5 mice in each experimental group was measured by IVIS 5 min after the IP injection of D-luciferin potassium. The luminescence was recorded before treatment on day 0, 4, 8, and 12. In order to perform the histological analysis, 1–3 mice in each experimental group were sacrificed at Day 14 and the tumors were excised, fixed in formalin, embedded in paraffin, and sectioned. Hematoxylin and eosin (H&E) staining was used for histological observations. All specimen evaluation was performed on an Olympus microscope using an ocular magnification of ×2.

#### Ewing’s sarcoma mouse models

For analyzing therapeutic efficacy for Ewing’s sarcoma, 18 athymic nude mice with SKES1 sarcoma cancer, 21 days after tumor implantation, were assigned into 3 groups, including saline control, free doxorubicin, and Lactate nanoparticle groups. 6 mice in each experimental group were intravenously injected once every 7 days with 200 μL of sterile saline suspension of free doxorubicin, and Lactate nanoparticle at the dose of 4 mg doxorubicin/kg. Injections of sterile saline at equivalent volume were given to mice once every 7 days as control. Mice were euthanized once the tumor reaches 1500 mm^3^. Tumor volumes were recorded after treatment on day 0, 3, 7, and 10. The luminescence was recorded before treatment on day 0, 5, 10, and 15.

#### Tumor dissociation into single-cell suspension

In order to perform single-cell RNA sequencing analysis, 9 days after treatment, 3 mice from each group were sacrificed and the tumors were excised. Dissected primary tumors were washed with cold RPMI 1640 media (Cytiva, SH30027.01), then minced into 1-mm cubes. Tumor cubes were then resuspended in RPMI 1640 media (Cytiva, SH30027.01) containing 1 mg/mL collagenase IV (Sigma-Aldrich, C5138-100MG) and 20 μg/mL DNase I (Sigma-Aldrich, 10104159001) before incubated for 30 min at 37°C. The tumor pieces were then ground and filtered through 70 μm cell strainers (Corning, 431751) to get single cells. Then the filtered cells were resuspended by Red Cell Lysis Buffer (eBioscience, 00-4300-54) and incubated for 5 min at room temperature to lyse red blood cells. Lysis was stopped by adding an equal volume of RPMI 1640 media (Cytiva, SH30027.01). Cells were washed twice with RPMI 1640 media (Cytiva, SH30027.01) and filtered through 70 cell strainers (Corning, 431751), then cells were ready for single-cell omics assays.

#### Single-cell omics assays

Cells from the tumor suspension were washed with cold FACS buffer (PBS, 2% BSA, 0.05% sodium azide). Fc receptors were blocked with Mouse TruStain FcX (BioLegend, 101319) at 1:50 dilution for 5 min at 4°C. Then, cells were incubated for 30 min at 4°C in the dark with a staining solution containing Total-seq anti-mouse Hashtag antibodies (BioLegend, 155861 for Hash 1, 155863 for Hash 2, 155865 for Hash 3) and AF647-labeled anti-CD45 (clone 30-F11, BioLegend, 103124) for leukocyte detection. The three replicates for each experimental condition were each labeled with a different Hashtag antibody. Subsequently, stained cells were conjugated with LIVE/DEAD Fixable Near-IR viability dye (Invitrogen, L34975) at 1:1000 dilution in PBS for 5 min at room temperature. Finally, cells were washed three times in cold cell media (RPMI, 10% FBS) before fluorescence-activated cell sorting (BD Biosciences, FACSAria Fusion). CD45^+^ sorting gates were drawn based on a fluorescence-minus-one control on the AF647 channel. Up to 13000 CD45^+^ live cells were sorted from each sample.

The Hashtag-labeled sorted cells from each replicate were pooled and partitioned into droplets for single-cell omics assays via Chromium Next GEM Single Cell 5′ Reagent Kits v2 (10x Genomics, 1000263). RNA-seq libraries were prepared according to manufacturer protocols. Libraries for Hashtagging were prepared via the 5′ Feature Barcode Kit (10x Genomics, 1000256). All libraries were quantified via the Qubit dsDNA HS Assay Kit (Invitrogen, Q32851), quality-checked for fragment sizes via high-sensitivity D5000 screentapes (Agilent, 5067–5592), pooled, and sequenced (Illumina, NextSeq 550).

#### Analysis methods

FASTQ files were generated using the CellRanger^130^ (v. 6.1.2) “mkfastq” command. Alignment, filtering, barcode counting, UMI counting, and demultiplexing was performed using the Cellranger “multi” command to retrieve sample matrices.

Downstream analysis was performed using the Seurat^131^ package (v. 4.0.3). Upon merging matrices from each sample, we filtered cells with greater than 15% mitochondrial reads and 40,000 unique molecular identifiers (UMIs). The matrix was normalized and the top 10,000 variable genes were taken as variable genes. We regressed out effects of mitochondrial percentage, UMI count, gene count, and cell cycle scores using the Seurat’s “ScaleData” function. We performed principal component analysis (PCA) using the top 100 principal components (PCs) and all variable genes. We then performed integration using the Harmony^132^ package (v. 0.1.0) using the top 100 PCs, the top 50 of which were used for Uniform Manifold and Projection (UMAP) dimensionality reduction.

Differentially expressed genes were determined using a Wilcoxon Rank-Sum test with Seurat’s “FindMarkers” with *p* values adjusted based on Bonferroni correction, and were used to annotate each cell cluster. We used the ClusterProfiler^133^ package (v.4.2.2) to perform gene set enrichment analysis (GSEA) using pathways from the MSigDB^134^ database. Gene module scores were calculated using Seurat’s “AddModuleScore” function using the genes from the “Goldrath_Eff_Vs._Memory_CD8_T cell_Up”^135^ and “GSE9650_Effector_Vs._Exhausted_CD8_T cell_Up” pathways.^136^ We used the Augur^85^ package (v. 1.0.3) to calculate cell-type prioritization area under the curve (AUC) scores separately between paired conditions.

Heatmaps were generated using the complexHeatmap^137^ package (v. 2.10.0). Dot plots and violin plots were generated using a combination of Seurat’s “DotPlot” and “VlnPlot” functions, ggsignif’s^138^ (v.0.6.2) “geom_signif” function, and ggplot2^139^ functions (v.3.3.6). Stacked violin plots were generated using code from method adopted by Ming Tang.

#### Histological analysis of combination treatment in breast cancer mouse models

In order to perform the histological analysis, all mice were sacrificed and the tumors were excised, fixed in formalin, embedded in paraffin, and sectioned. Anti-Granzyme B antibody, and anti-TOX antibody staining was used for histological observations. All specimen evaluation was performed on an Olympus microscope using a magnification of ×400 (Inverted microscope; Nikon ECLIPSE Ti2). Areas of normal and benign breast were excluded from the final assessment. The number of immunostained-positive cells was assessed by two independent observers. The immunostained cells were considered positive when there was homogeneous and clearly visible brown staining and negative if this staining was absent. The numbers of effector or exhausted cell were obtained by calculating the number of Granzyme B or TOX-positive cells in a microscopic grid, 1 × 1 mm in size (1 mm^2^). Each datum was counted in a minimum of four randomly selected areas in immunohistochemistry staining images. The counts are expressed as the mean number of cells per square millimeter. Control slides (minus primary antibody) were assessed for non-specific binding before assessing the percentage of tumor cell binding the anti-Granzyme B and anti-TOX antibodies.

#### Toxicity of different treatments *in vivo*

For analyzing long term toxicity with different treatments, 35 CD1 mice (8 weeks, female) were assigned into 7 groups, including saline control, free doxorubicin, doxorubicin-loaded pH-responsive nanocarrier, doxorubicin loaded lactate-responsive nanoparticles, free SR-717, SR-717 loaded lactate-responsive nanoparticles, and Combo groups. 5 mice in free doxorubicin, doxorubicin-loaded pH-responsive nanocarrier, and doxorubicin loaded lactate-responsive nanoparticles groups were intravenously injected once per week with 200 μL of sterile saline suspension of free doxorubicin, doxorubicin-loaded pH-responsive nanocarrier, and doxorubicin loaded lactate-responsive nanoparticles at the dose of 4 mg doxorubicin/kg, respectively. 5 mice in free SR-717 group and SR-717 loaded lactate-responsive nanoparticles group were intravenously injected once per week with 200 μL of sterile saline suspension of free SR-717 and SR-717 loaded lactate-responsive nanoparticles at the dose of 4 mg SR-717/kg. 5 mice in Combo group were intravenously injected once per week for two weeks with 200 μL of sterile saline suspension of SR717 loaded lactate-responsive nanoparticles at the dose of 4 mg SR-717/kg. 2 days after that, mice in Combo group were intraperitoneally injected once every 3 days for 3 dose with 200 μL of sterile saline suspension of antiPD1 at the dose of 12.5 mg antiPD1/kg. Injections of sterile saline at equivalent volume were given to mice once per week as control. Mice weight was recorded 0, 2, 7, 9, 12, 16, 21, and 28 days after treatment.

### Quantification and statistical analysis

The figure legends provide information on group sizes, mean and error bars. Statistical analysis was performed using Excel or OriginLab software. Comparison between quantitative data were conducted using the unpaired or paired Student’s t test, Mann-Whitney U-test, or Dunnett’s t-test, where appropriate. All *p* values were two-tailed and *p* values of 0.05 or less were considered to be statistically significant (∗*p* < 0.05, ∗∗*p* < 0.01, ∗∗∗*p* < 0.001).

## Supplemental information

Document S1. Figures S1–S7

Document S2. Article plus supplemental information
