# Supplementary material for: Enabling tumor-specific drug delivery by targeting the Warburg effect of cancer
Source: Cell Rep Med. 2025 Jan 13;6(1):101920. doi: 10.1016/j.xcrm.2024.101920 (PMC11866520; doi:10.1016/j.xcrm.2024.101920)
Supplement: Document S1. Figures S1–S7 [file mmc1.pdf]

Cell Reports Medicine, Volume *6*

Supplemental information

Enabling tumor-specific drug delivery

by targeting the Warburg effect of cancer

Jian Zhang, Tony Pan, Jimmy Lee, Sanja Goldberg, Sarah Ann King, Erting Tang, Yifei Hu, Lifeng Chen, Alex Hoover, Linyong Zhu, Oliver S. Eng, Benjamin Dekel, Jun Huang, and Xiaoyang Wu

Supplementary Figure 1

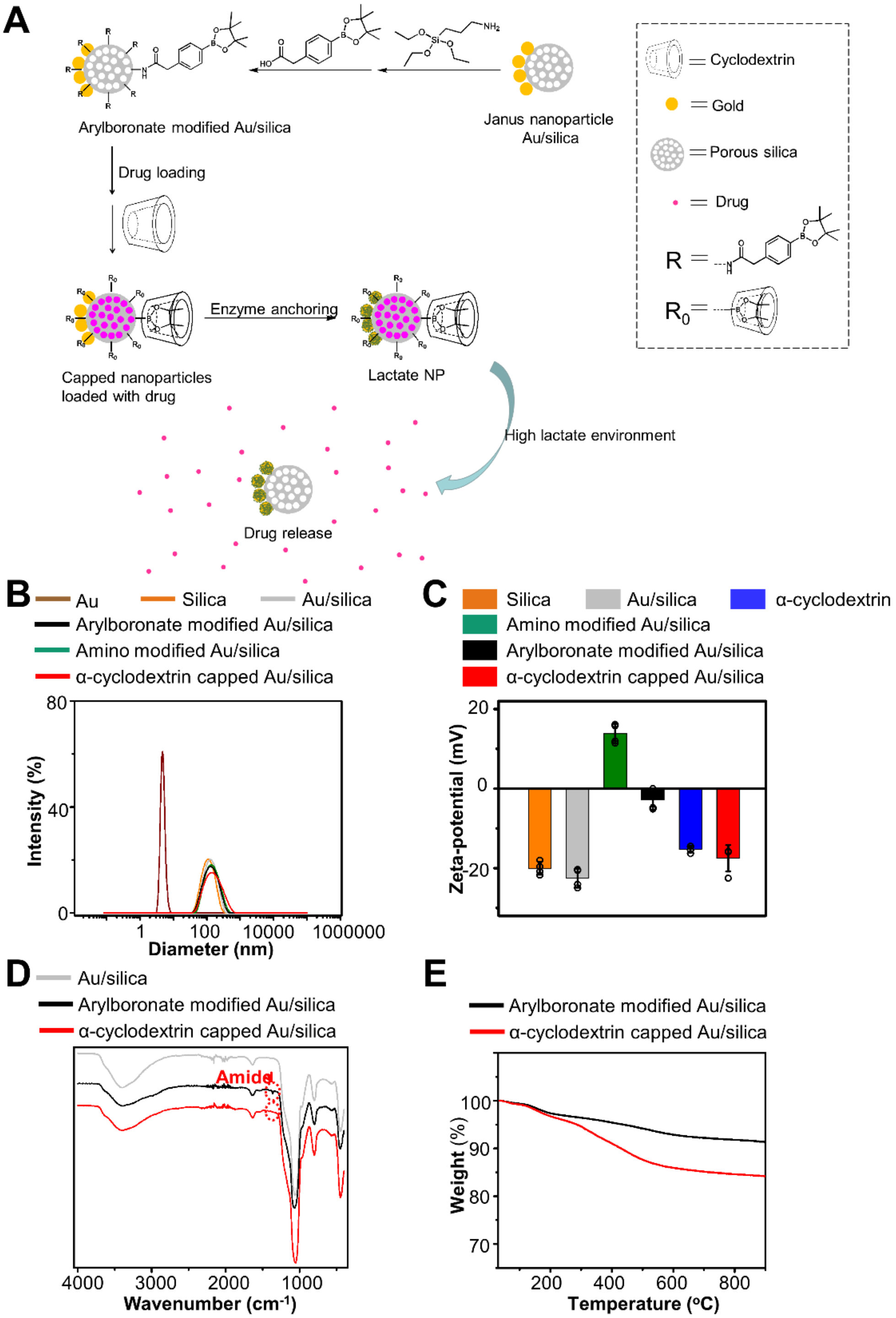

Supplementary Figure 1: Development of lactate-responsive Janus nanoparticles for drug delivery. Related to Figure 1. (A) Schematics for fabrication of the lactate-responsive Janus particles. The surface of mesoporous silica nanoparticle is functionalized with arylboronate derivatives and capped by host–guest complexation with α-cyclodextrin. With anchored lactate oxidase on the surface of Au, lactate in the tumor microenvironment will lead to the production of hydrogen peroxide, which can lead to self-immolation of

arylboronate. This reaction can uncap mesoporous silica nanoparticle and release of the drug inside. R: Arylboronate derivative. R_0: Host–guest complex with α-cyclodextrin. **(B-C)** Characterizations of the nanoparticles by dynamic light scattering (B) and ζ-potential analysis (C). n = 4. Data are presented as mean ± SD. All error bars represent SD. **(D)** Fourier transform infrared (FT-IR) spectroscopy was employed to characterize various functional groups on the nanoparticles. After the conjugation of arylboronate derivative to Au/silica, the amide bond stretching in arylboronate modified Au/silica can be observed at 1350 cm^{-1}. After the coupling with α-cyclodextrin, the amide bond stretching was bended and masked by the –C-H stretching from α-cyclodextrin. **(E)** Quantification of the weight ratio of conjugated arylboronate derivatives and coupled α-cyclodextrin of the nanoparticles were determined by thermogravimetric analysis (TGA).

Supplementary Figure 2

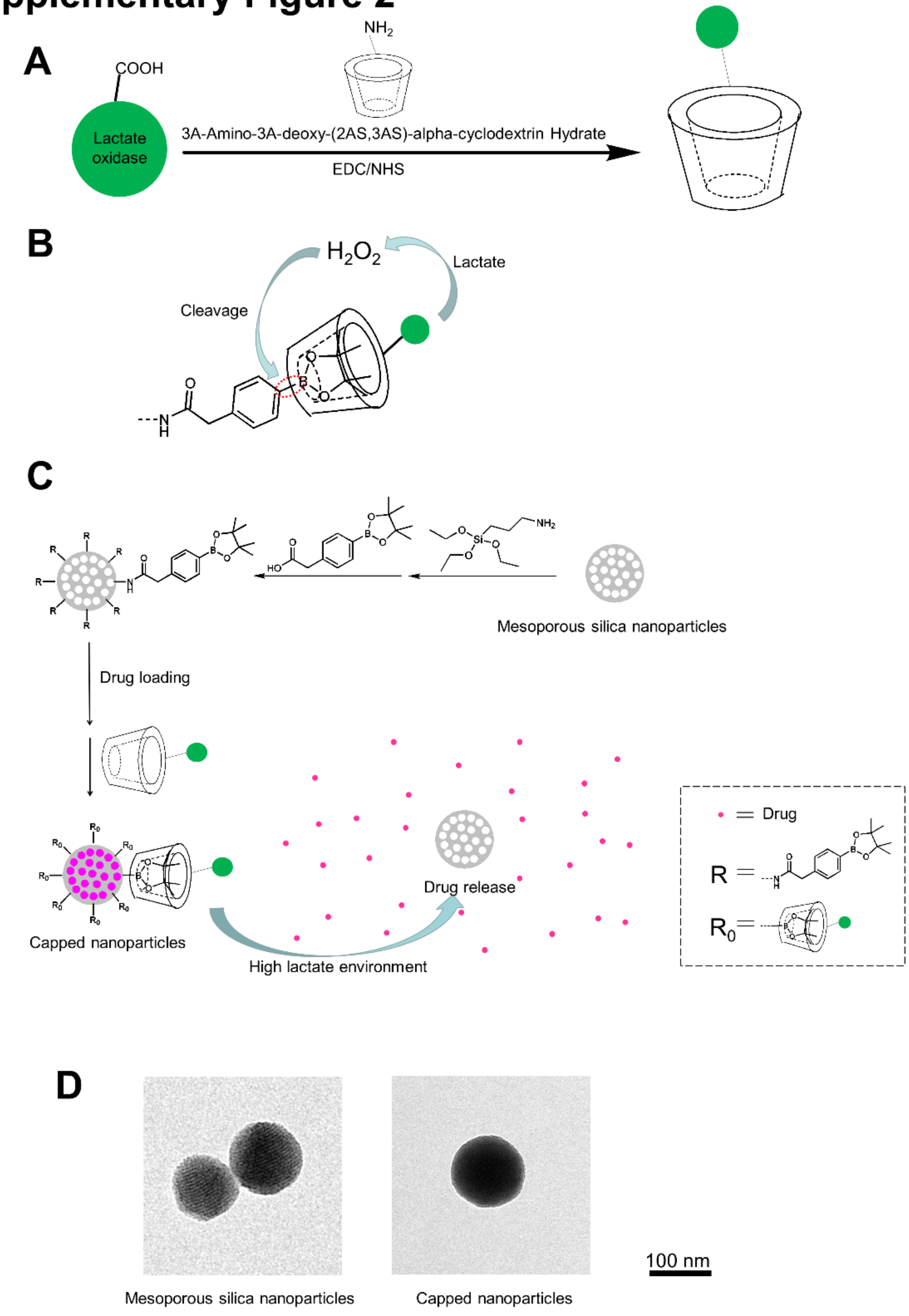

Supplementary Figure 2: **Alternative strategy for engineering lactate-responsive drug carriers. Related to Figure 1. (A)** Lactate oxidase can be covalently linked with 3A-Amino-3A-deoxy-(2AS,3AS)-α-cyclodextrin, the capping molecules. **(B)** With the presence of lactate, lactate oxidase produces hydrogen peroxide, which induces the self-immolation reaction of arylboronate derivatives. **(C)** The surface of mesoporous silica nanoparticles is capped by host–guest complexation with enzyme functionalized

cyclodextrin. With the lactate oxidase on the surface of the nanoparticles, lactate in the tumor microenvironment can lead to uncapping and the release of the drug inside. R: Arylboronate derivative. R_0: Host–guest complex with lactate oxidase linked 3A-Amino-3A-deoxy-(2AS,3AS)-α-cyclodextrin. **(D)** Representative TEM images of the mesoporous silica nanoparticles (left panel) and the capped nanoparticles with enzyme-functionalized α-cyclodextrin (right panel).

Supplementary Figure 3

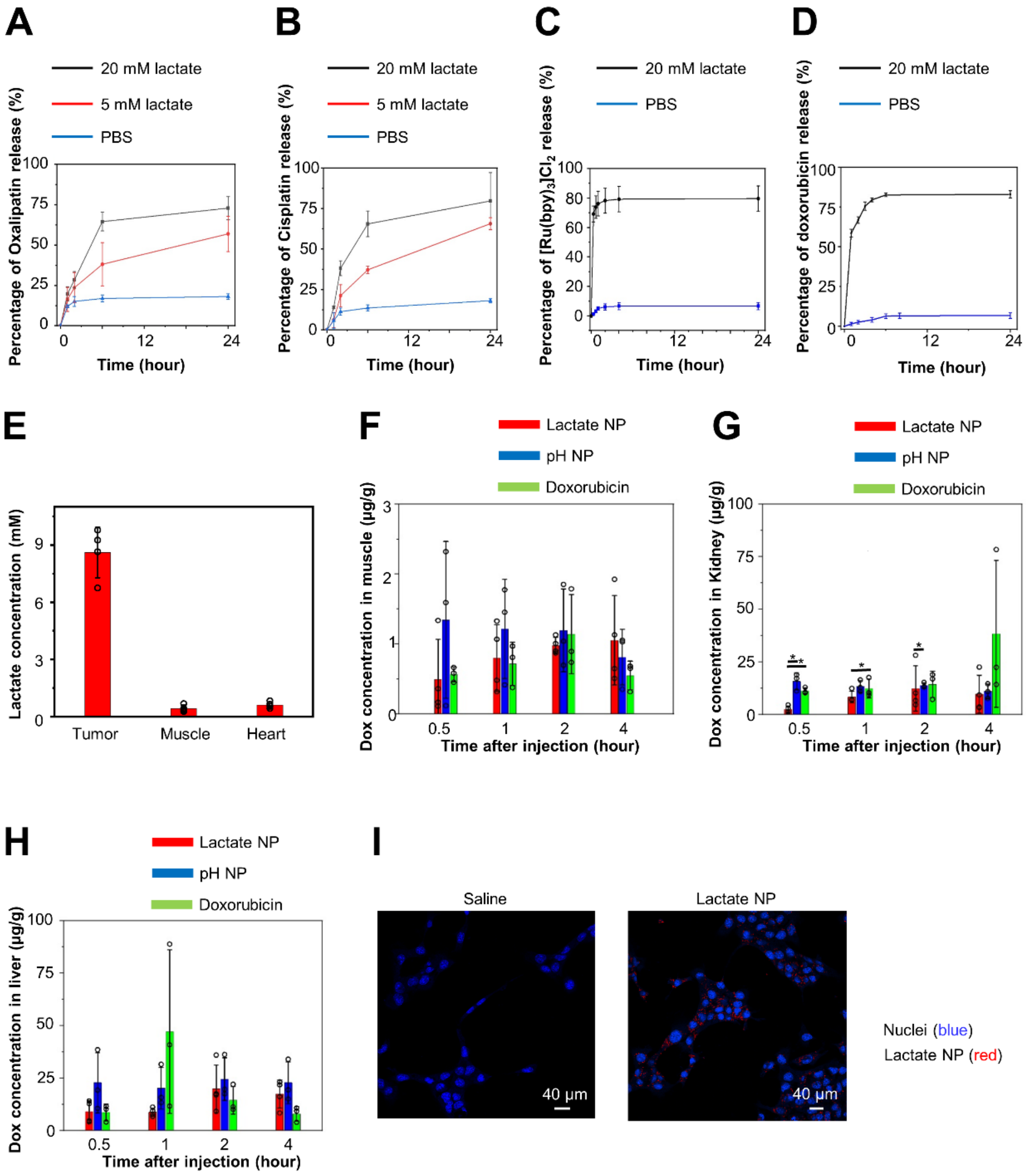

Supplementary Figure 3: **Lactate-inducible drug release *in vitro* and enhanced tumor-specific drug delivery *in vivo*. Related to Figures 2 and 3. (A-C)** Release kinetics of Oxaliplatin (A), Cisplatin (B), and tris(bipyridine)ruthenium(II) chloride (C) from the Janus nanoparticles at different concentrations of lactate. **(D)** Release kinetics of doxorubicin from non-Janus nanoparticles (direct enzyme functionalization with α-cyclodextrin) at different concentrations of lactate. n = 3. Data are presented as mean ± SD. All

error bars represent SD. **(E)** Lactate concentration in tumor or health tissues were determined by biochemical enzymatic assay kit . n = 4 independent samples. All error bars represent SD. **(F-H)** The bio-distribution of doxorubicin in muscle (F), kidney (G), and liver (H) was determined by liquid chromatography/mass spectrometry. n = 3. Data are presented as mean ± SD. All error bars represent SD. **(I)** Confocal fluorescence imaging of doxorubicin-loaded nanoparticles in treated 4T1 cells *in vitro*. Nuclei are stained with DAPI and shown in blue. Doxorubicin-loaded Janus nanoparticles are shown in red.

Supplementary Figure 4

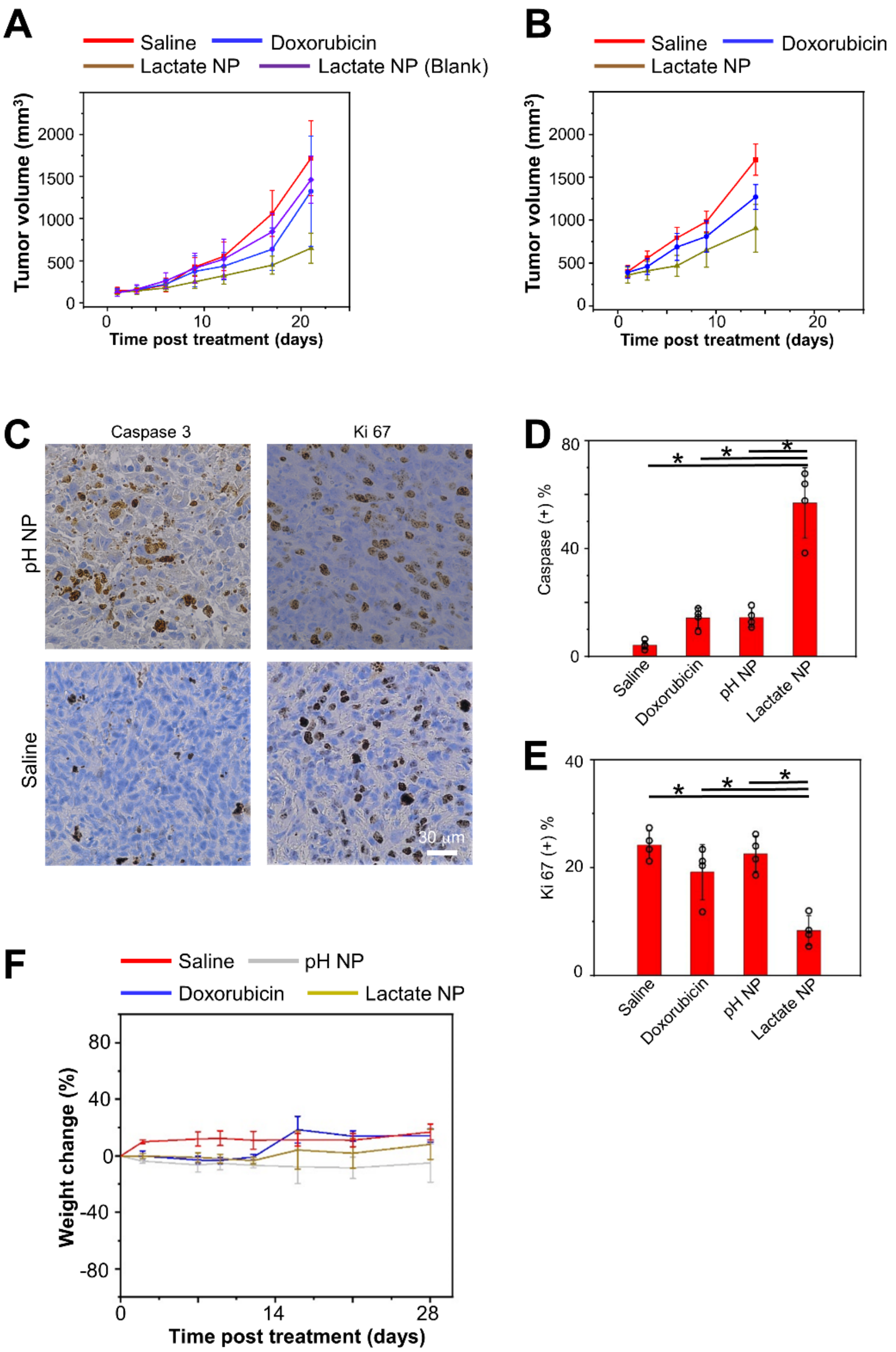

Supplementary Figure 4: Lactate-responsive drug carriers enhance therapeutic efficacy of chemotherapy *in vivo*. Related to Figure 4. (A-B) Quantification of TNBC tumor growth with different initial tumor size 130 mm^3 (A) and 385 mm^3 (B) upon treatments. n = 4 independent samples. Data are presented as mean ± SD. All error bars represent SD. **(C)** Cell proliferation and apoptosis in tumor tissue were determined by immunohistochemistry for Ki67 and Caspase3 respectively upon treatment with

doxorubicin-loaded pH-responsive nanocarriers (pH NP) and saline control. **(D-E)** Quantification of tumor cell proliferation and apoptosis upon different treatments as indicated. n = 4. Data are presented as mean ± SD. All error bars represent SD. *: $p<0.05$ (student's t-test). **(F)** No significant weight change after doxorubicin treatment with or without drug carriers. n = 5. Data are presented as mean ± SD. All error bars represent SD.

Supplementary Figure 5

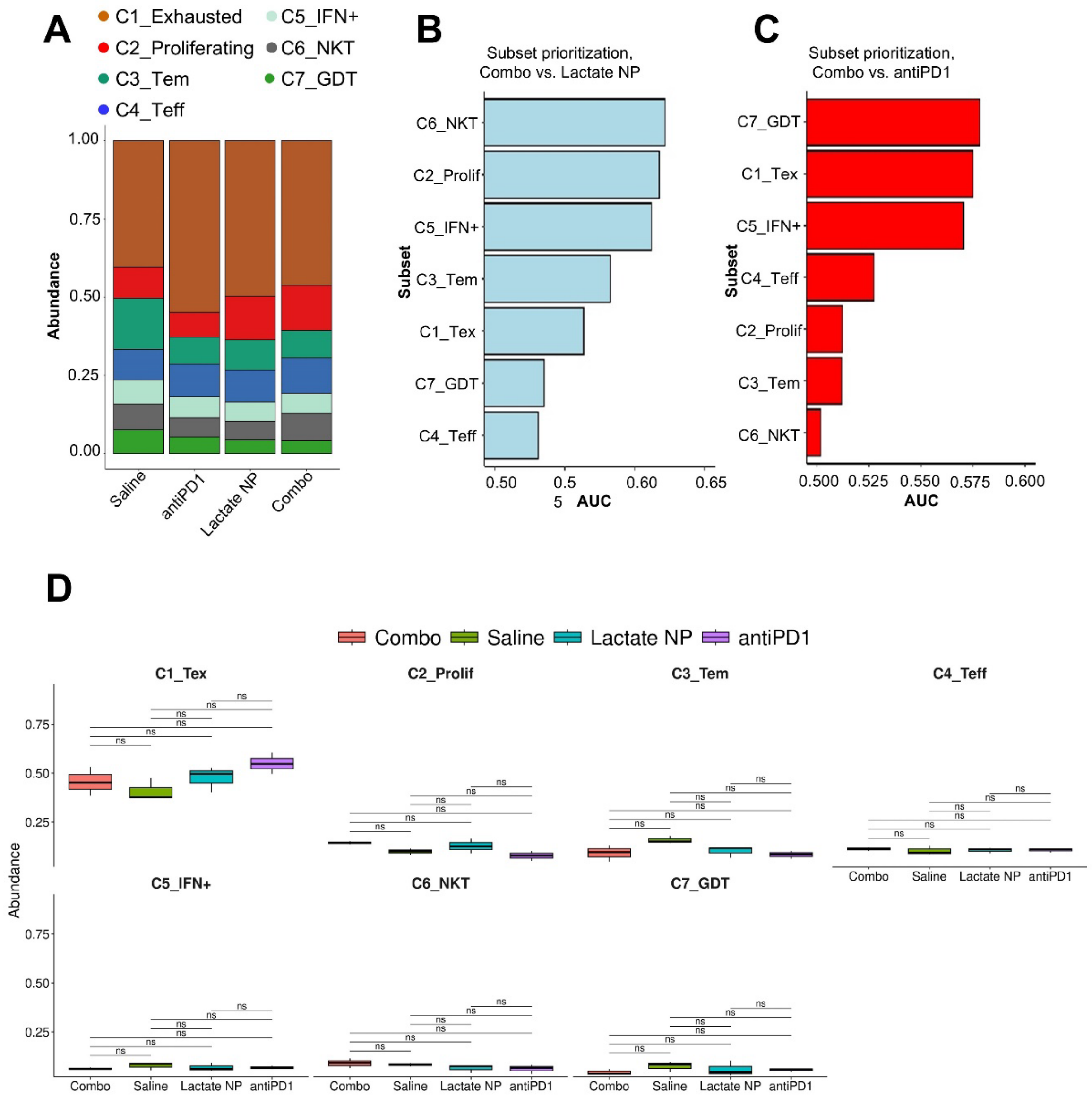

Supplementary Figure 5: Delivery of STING agonist with the lactate-responsive nanocarrier can enhance the efficacy of immunotherapy for breast cancer. **Related to Figure 6.** Proliferating and Exhausted clusters were highly ranked in distinguishing the combo treated CD^{8+} T cells from the monotherapy groups. **(A)** Abundance ratio of the cell clusters. **(B-C)** Line plots of Augur-derived AUC scores illustrating cell-type prioritization between Combo treated and SR-717 loaded lactate responsive nanoparticles treated CD^{8+} T cells (B), and between Combo treated and antiPD1 treated CD^{8+} T cells (C). **(D)** Box plots of the abundance of each CD^{8+} T cell subpopulation found in each treatment group (n=3 per group). Adjusted p-values were computed by the Wilcoxon Rank-sum test with Bonferroni correction. NS denotes adjusted an adjusted p-value > 0.05.

Supplementary Figure 6

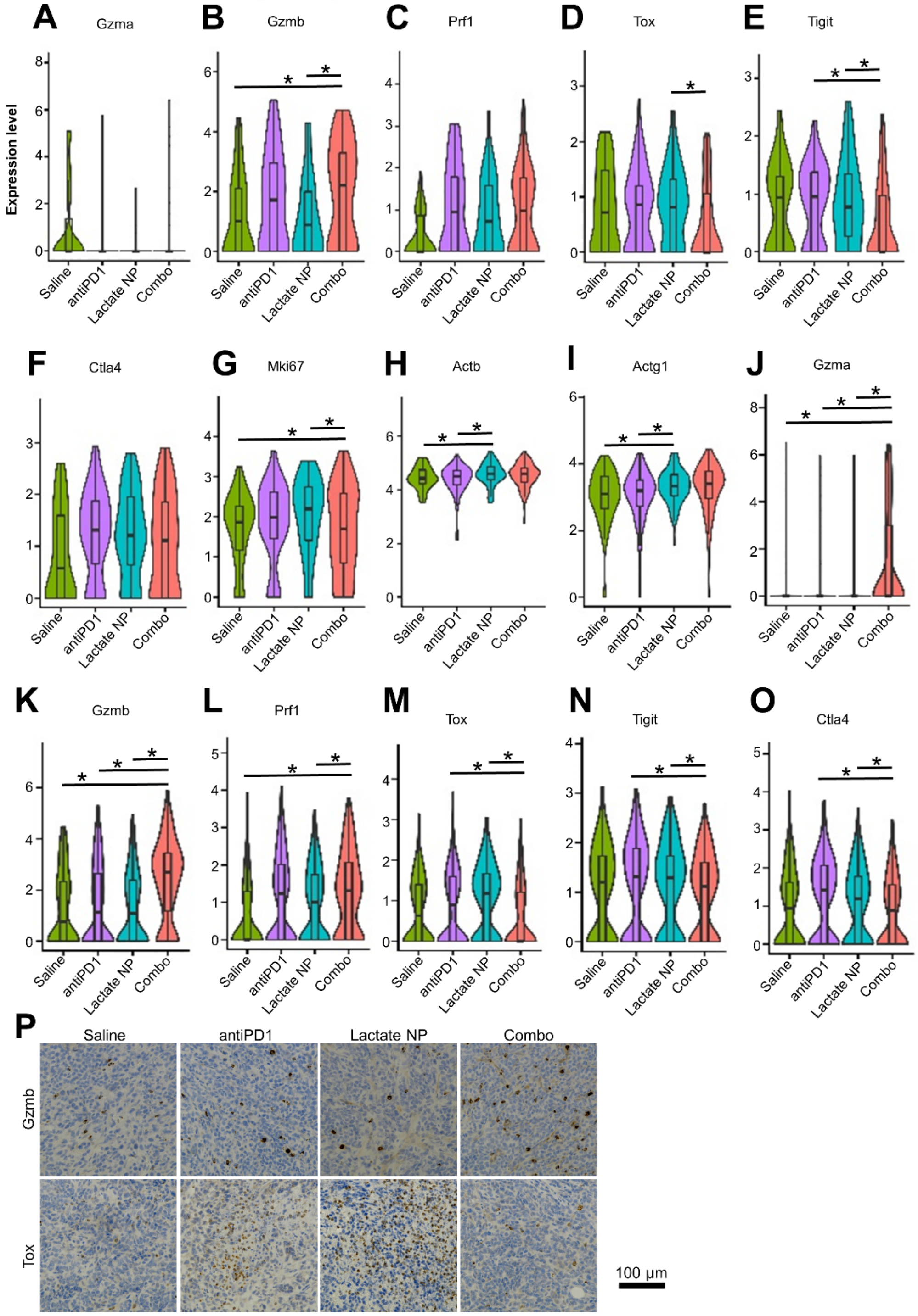

Supplementary Figure 6: Delivery of STING agonist with the lactate-responsive nanocarrier can alter the immune-microenvironment of breast cancer. **Related to Figure 7.** Upregulation of effector function in CD^{8+} T cells were found in the Combo treated tumor, while the SR-717 loaded lactate responsive nanoparticles treated and the antiPD1 treated CD^{8+} T cells expressed lower effectorness and higher exhaustion. **(A-I)** Comparisons of functional gene expression across conditions in proliferating

CD8+ T cells. Violin plots contrasting expression of effectorness Gzma (A), Gzmb (B), and Prf1 (C) in proliferating CD^{8+} T cells. Violin plots contrasting expression of exhaustion genes Tox (D), Tigit (E), and Ctla4 (F) in proliferating CD^{8+} T cells. Violin plots contrasting expression of proliferation genes Mki67 (G), Actb (H), and Actg1 (I) in proliferating CD^{8+} T cells. **(J-O)** Comparisons of functional gene expression across conditions in exhausted CD8+ T cells. Violin plots contrasting expression of effectorness genes Gzma (J), Gzmb (K), and Prf1 (L), and exhaustion genes Tox (M), Tigit (N), and Ctla4 (O) in exhausted CD8+ T cells. **(P)** Immunohistochemical staining for paraffin-embedded breast cancer sections for detection of tumor-infiltrating granzyme B+ and TOX+ cells in different treatment groups. In the combo treatment group, Gzmb+ cells were significantly accumulated whereas TOX+ cells were reduced.

Supplementary Figure 7

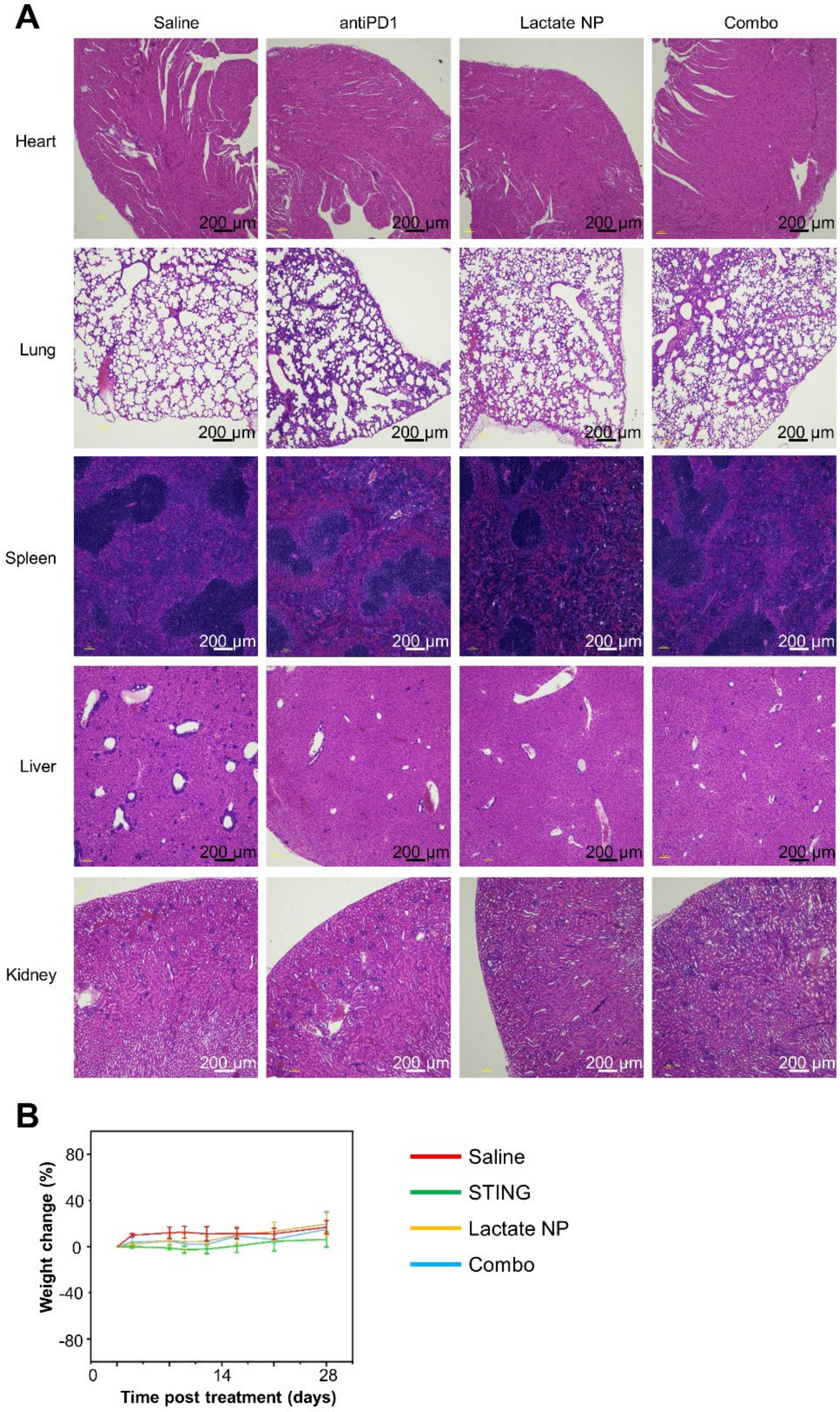

Supplementary Figure 7: Immunotherapy with the lactate-responsive drug carriers does not lead to significant toxicity *in vivo*. Related to Figure 7. **(A)** Potential pathology in different organs were determined by tissue histology upon different treatments as indicated. **(B)** Body weight change upon different treatments as indicated. n = 5. Data are presented as mean ± SD. All error bars represent SD.
